# Acupuncture Inhibits Morphine Induced-Immune Suppress via Antioxidant System

**DOI:** 10.1155/2022/7971801

**Published:** 2022-10-22

**Authors:** Rong Jie Zhao, Dae Geon Lee, Chan Sik Park, Chae Ha Yang, Hee Young Kim, Mi Young Lee, Chang-Hyun Song, Il Je Cho, Sang Chan Kim, Sae Kwang Ku, Bong Hyo Lee

**Affiliations:** ^1^Department, of School of Mental Health, Qiqihar Medical University, Qiqihar, Heilongjiang Province 161006, China; ^2^Department of Anatomy and Histology, College of Korean Medicine, Daegu Haany University, Gyeongsan 38610, Republic of Korea; ^3^Research Center for Herbal Convergence on Liver Disease, Daegu Haany University, Gyeongsan 38610, Republic of Korea; ^4^Department of Acupuncture, Moxibustion and Acupoint, College of Korean Medicine, Daegu Haany University, Daegu 42158, Republic of Korea; ^5^Department of Physiology, College of Korean Medicine, Daegu Haany University, Daegu 706-828, Republic of Korea; ^6^Department of Physiology, Yonsei University College of Medicine, Seoul 03722, Republic of Korea; ^7^Department of Physical Therapy, College of Biomedical Science, Daegu Haany University, Gyeongsan 38610, Republic of Korea; ^8^Department of Herbal Formula, College of Biomedical Science, Daegu Haany University, Gyeongsan 38610, Republic of Korea

## Abstract

**Objectives:**

A powerful analgesic called Morphine causes addiction behaviors and immune suppression as a potential oxidative stressor. Acupuncture showed to inhibit oxidative stress-induced hepatic damage, regulate reactive oxygen species, and attenuate morphine addiction behaviors. Therefore, we investigated the potential effects of acupuncture on morphine-induced immune suppression.

**Materials and Methods:**

Rats received morphine intravenously through implanted catheters for 3, 7, or 21 days to determine the optimal condition for morphine-induced immune suppression. Second, we examined whether intravenous (iv.) or intraperitoneal (ip.) administration produced different results. Third, the effects of acupuncture in rats who received morphine for 21 days were investigated. Spleen and submandibular lymph node (S-LN) weights and natural killer (NK) cell activity were measured, and the white pulp diameter, total and cortical spleen thicknesses, and the number of lymphoid follicles in S-LNs were examined. The number of immunoreactive cells was also measured.

**Results:**

Decreased organ weights and increased atrophic changes were observed as morphine-induced immune suppression. However, dose-dependent increased immune suppression was not observed between 5.0 mg/kg and 10.0 mg/kg of morphine. And, 3-day withdrawal did not affect. Similar histopathological findings were observed in 5.0 and 10.0 ip. rats when compared to equal dosages of iv., respectively. The morphine induced-immune suppression evidenced by spleen and left S-LN weights, splenic NK cell activities, histopathological findings, and the immunoreactive cell number were normalized by acupuncture.

**Conclusion:**

These results indicate that acupuncture inhibits morphine-induced immune suppression, maybe via antioxidative action.

## 1. Introduction

Addiction is a serious problem and has negative health, social, economic, and cultural effects [1–3]. The most common drugs abused are opiates including opium, codeine, morphine, and heroin [1]. Morphine, a natural alkaloid found in opium poppy [4], has been frequently used to treat severe pain due to its powerful analgesics and sedative effects [5, 6]. Morphine suppresses the affective reaction to pain by inhibiting transmission of pain impulses, especially in the spinal cord, and through modulation of central neural circuits in the brain. However, morphine causes adverse effects when improperly prescribed [7], including respiratory, cardiovascular, gastrointestinal, or psychiatric problems [6, 8–10]. In addition, morphine acts as a potential oxidative stress-causing agent [7], and induces oxidative stress-related hepatic damage [11–13]. Some studies with innate immune cells from animals and humans and animal in vivo studies have shown that opiate abuse impairs innate immunity and is responsible for increased susceptibility to bacterial infection [14, 15]. Morphine weakens immune system activity and inhibits immune response in the spleen, thymus, and lymph nodes [1, 4]. Opioid causes spleen atrophy [16, 17] and reduces the number of natural killer (NK) cells [18, 19] B and T lymphocytes [20, 21].

Immune functions are indispensable because they are the host defenses against infection and cancer and play a crucial role in maintaining health [22]. Declining immune function that occurs due to aging, chronic illnesses, physical and mental stress, or unhealthy lifestyles has been a major health problem [22–24], and therefore, modulation of the immune function has attracted great interest [25, 26]. However, many of the available immunomodulators such as levamisole, glucans, telerones, L-fucose, and *Corynebacterium parvum*, showed side effects such as fever, neutropenia, leucopenia, and allergic reactions [26, 27]. Hence, efforts to find better agents and evaluate their immunomodulatory potential have been performed [26].

Certain nutrients play a crucial role in the maintenance of optimum immune responses, and both deficiency and excessive intake can adversely affect the number and activity of the immune cells [26]. The underlying mechanism by which nutrients support the immune system is via the provision of antioxidants. Immune cells such as T-cells, NK cells, and T-helper cells are characterized by excessive levels of reactive oxygen species (ROS), which kill ingested pathogens. In addition, immune cell membranes are enriched with polyunsaturated fatty acids that are susceptible to ROS-mediated damage [26, 28]. Therefore, supplementation of nutrients with antioxidant properties such as carotenes, vitamin E, vitamin C, zinc, selenium, and polyphenols may quench these free radicals and influence several components of the immune system [26, 29].

Acupuncture, one of the main therapies of traditional Eastern Asian medicine, has been considered an alternative treatment in western countries for many diseases, including pain, asthma, and neuropsychiatric diseases [30–32]. In drug addiction, acupuncture had shown normalizing effects [33–35]. Furthermore, for morphine addiction, acupuncture showed potential therapeutic effects in diverse situations [36–39].

Notably, experimental and clinical studies confirmed that acupuncture influence the immune system [40, 41]. In addition, acupuncture showed antioxidant properties [42–44], especially in morphine-induced liver injury [13], and regulated ROS levels [45]. Thus, we hypothesized that acupuncture could regulate morphine-induced immune suppression and investigated possible mechanisms, based on the previous studies.

## 2. Methods

### 2.1. Animals

Male Sprague-Dawley rats (Daehan Animal, Seoul, Korea) weighed 270–300 g were used when the study began. Housing conditions were temperature (22 ± 2°C), humidity (60 ± 5%), and 12 h light-dark cycle (turn on at 7 : 00 pm). They freely accessed food and water and were acclimated to the experimental environment before the experiment. Experimental procedures were approved by the Institutional Animal Care and Use Committee at Daegu Haany University.

### 2.2. Surgery

To mimic the same condition with humans of intravenous morphine administration, animals were given catheter implantation surgery. Chronic silastic catheters (Dow Corning, Midland, MI, USA; 0.02 inch ID × 0.037 inches OD) were implanted into the right jugular vein under anesthesia with pentobarbital (50 ㎎/㎏, i. p.) and fixed using Mersilene mesh (Ethicon Inc., Somerville, NJ, USA) [46]. The catheter was exteriorized at the back of the animals using a 22 gauge guide cannula (Palstics One, Roanoke, VA, USA) through the skin incision. Silastic tubing and guide cannulae were embedded in dental cement and secured with Prolene mesh. 0.2 ㎖ solution of saline containing heparin (30 U/㎖) was flushed into the catheter daily to maintain patency during recovery from surgery.

### 2.3. Morphine Treatment

Morphine hydrochloride (JEIL Pharmaceutical Co., Daegu, Korea) or saline was given according to the experimental design.

### 2.4. Experimental Design

#### 2.4.1. Morphine Effect on Immunity

Rats of the following groups received each treatment intravenously. Normal (intact) group: vehicle (saline); M 0.1 group: morphine 0.1 mg/kg; M 1.0 group: morphine 1.0 mg/kg; M 5.0 group: morphine 5.0 mg/kg; M 10.0 group: morphine 10.0 mg/kg. Each dose was administered for each of the following 4 periods. 3 days (3D), 7 days (7D), 21 days (21D), and 21D withdrawal of 3 following days (21D3DW). These dosages were selected based on the clinical treatment for humans and the previous animal studies about intravenous morphine administration [36–39], and we also assigned lower dose of 0.1 mg/kg to examine if morphine induces immune decrease at a low dose Each group *n* = 5.

#### 2.4.2. Comparison of the Effects of Administration Route

To investigate if there is a significant difference between the effects of intravenous (i. v.) and intraperitoneal (i. p.) administrations on immunity, other animals were subjected to morphine for 21D after assignment to the following 4 groups. M 5.0 i. v. group; M 10.0 i. v. group; M 5.0 i. p. group; M 10.0 i. p group. *n* = 5 (M 5.0) or 6 (M 10.0).

#### 2.4.3. Acupuncture Effects on Immune Suppression by Morphine

The different rats were assigned to the following 5 groups to investigate the effects of acupuncture on the decrease of immunity induced by morphine treatment. Normal group (*n* = 9): vehicle; Control group (*n* = 7): morphine; HT7 group (*n* = 9): morphine + acupuncture at HT7; SI5 group (*n* = 9): morphine + acupuncture at SI5; LI5 group (*n* = 9): morphine + acupuncture at LI5. Animals were given morphine or vehicle for 21 days intravenously.

### 2.5. Acupuncture

Acupuncture groups received acupuncture treatment at each acupoint bilaterally. HT7 is located on the transverse crease of the wrist of the forepaw, radial to the tendon of the muscle flexor carpi ulnaris [46, 47]. SI5 is located on the posteromedial aspect of the wrist in the depression between the triquetral bone and the styloid process of the ulna [47, 48]. LI5 is located on the posterolateral aspect of the wrist, at the radial side of the dorsal wrist crease, distal to the radial styloid process, in the depression of the anatomical snuffbox [46, 47]. The locations of acupoints followed the anatomical structures and were equivalent to those in human as described in the previous studies [38, 46].

Acupuncture was performed once a day for 1 min immediately after morphine by one researcher. A stainless-steel needle (diameter 0.18 mm, length 8 mm, Dongbang Acupuncture Inc., Chingdao, China) was inserted vertically into a depth of 2–3 mm, and was bidirectionally twisted for stimulation [38]. Rats received acupuncture in awaken state with a slight movement restriction. Daily handling was given for 2–3 min to minimize the stress from the movement restriction. The normal and control groups received the same treatment with acupuncture groups without needle stimulation.

### 2.6. Organ Weight Measurements

At sacrifice, the weights of individual spleen and left submandibular lymph node (S-LN) were measured at *g* levels (absolute wet-weights) using an automatic electronic balance (XB320 M, Precisa Instrument, Zuerich, Switzerland), and to reduce the differences between individual body weights, the relative weights (% of body weights) were also calculated using body weight at sacrifice and absolute weight as Relative Organ Weights (%) = [(Absolute spleen or S-LN wet-weights/Body weight at sacrifice) × 100].

### 2.7. Gross Findings

At sacrifice, any abnormal gross findings were recorded with digital images, and were subdivided into four degrees: 3+ Severe, 2+ moderate, 1+ slight, 0+ not detected-normal appearance [49].

### 2.8. Histopathology

At sacrifice, samples from spleen and left S-LN were fixed in 10% neutral buffered formalin (NBF). Equal regions of individual spleen and S-LN were crossly trimmed as one part in each organ, and all crossly trimmed spleen and S-LN parts were re-fixated in 10% NBF for 24 h. Then paraffin embedding blocks were prepared using an automated tissue processor (Shandon Citadel 2000, Thermo Scientific, Waltham, MA, USA) and embedding center (Shandon Histocentre 3, Thermo Scientific, Waltham, MA, USA), and 3–4 *μ*m sections were prepared using automated microtome (RM2255, Leica Biosystems, Nussloch, Germany). Representative sections were stained with HE for general histopathology [50, 51], and individual cross-trimmed spleen and S-LN tissues were light microscopically observed (Model Eclipse 80i, Nikon, Tokyo, Japan). To more detail changes, the total splenic thicknesses (mm/central regions), white pulp thickness (*μ*m/white pulps) and numbers (white pulps/mm^2^ of spleen), total and cortex thicknesses of S-LN (*μ*m/central regions), lymphoid follicle numbers (Follicles/mm^2^ of S-LN) were calculated using an automated image analyzer (iSolution FL ver 9.1, IMT i-solution Inc., Vancouver, Canada), respectively [50, 51].

### 2.9. Splenic NK Cell Activity Calculation

Splenic NK cell activities were measured using a standard ^51^Cr release assay [50, 52]. Briefly, splenocytes were collected from each animal. Spleen 10∼20 mg were separated and washed by RPMI-1640 medium, twice at 4°C. Homogenates were prepared using a bead beater (Taco^TM^Pre, GeneResearch Biotechnology Corp., Taichung, Taiwan) and an ultrasonic cell disruptor (KS-750, Madell Technology Corp., Ontario, CA, USA). Prepared splenic NK cells disrupted mechanically by maceration through a wire mesh (Mesh No. 100, Sigma-Aldrich, St. Louise, MO, USA) wetted with RPMI-1640 medium. The debris was allowed to settle, and the cell suspension was pelleted by centrifugation. RBCs were lysed by resuspending the pellet in cold 1% ammonium oxalate and incubating on ice for 10 min. The cells were pelleted and washed twice with HBSS (Hanks Balanced Salt Solution; Gibco BRL, Grand Island, NY, USA). Splenocytes were cultured overnight in Dulbecco's Modified Eagle Medium (Invitrogen, Grand Island, NY, USA) in the absence or presence of recombinant IL-2 (1000 IU/mL; Proleukin Chiron, Emeryville, CA, USA). The HTLA-230 neuroblastoma target cells were labeled for 2 hrs with Na_2_^51^CrO_4_ (100 *μ*Ci/1 × 106 cells) (ICN Biomedicals, Asse, Belgium). Target cells were then incubated for 6 h at 37°C with splenocytes as effector cells. The effector target cell ratio was 100 : 1. Supernatants were collected, and the amount of radioactivity released into the supernatants were counted with a gamma counter (Cobra 5002; Canberra Packard, Meriden, CT, USA). The percentage of specific target cell lysis was calculated as follows:

  Equation (2). % Specific ^51^Cr Release (NK Cell Activities). = [(Exp − S)/(M − S)] 100% (Where, Exp is the observed released ^51^Cr value, S is the spontaneously released ^51^Cr value, and M is the maximum released ^51^Cr value).

### 2.10. Immunohistochemistry

The changes of immunoreactivities in the spleen and S-LN against markers of T cell subsets-CD3, CD4, CD8, and Foxp3, general and hematopoietic stem cells-CD34 and CD45, and immune-relatedcytokines-iNOS, TNF-*α*, IFN-*γ*, IL-1*β*, IL-2, IL-4, IL-6, IL-10, and IL-12A were observed using purified primary antibodies (Table 1) with ABC (Avidin-biotin-peroxidase complex) and peroxidase substrate kit (Vector Labs, Burlingame, CA, USA). Briefly, endogenous peroxidase activity was blocked by incubating in methanol and 0.3% H_2_O_2_ for 30 min and nonspecific binding of immunoglobulin was blocked with normal horse serum blocking solution for 1 h in humidity chamber after heating (95–100°C) based epitope retrievals in 10 mM citrate buffers (pH 6.0) [50, 53]. Primary antisera were treated overnight at 4°C in a humidity chamber, and then incubated with biotinylated universal secondary antibody and ABC reagents for 1 h. Finally, sections were reacted with peroxidase substrate kit for 3 min. All sections were rinsed in 0.01 M phosphate-buffered saline 3 times, between each step.

### 2.11. Histomorphometry

Total splenic thicknesses (*μ*m/central regions), white pulp thickness (*μ*m/white pulps) and numbers (white pulps/mm^2^ of spleen), total and cortex thicknesses of S-LN (*μ*m/central regions), lymphoid follicle numbers (Follicles/mm^2^ of S-LN) were calculated using an automated image analyzer (*i*Solution FL ver 9.1, IMT *i*-solution Inc., Vancouver, British Columbia, Canada), respectively [50, 51]. In addition, the cells occupied over 20% of immunoreactivities, and the density of each antibody for CD3, CD4, CD8, Foxp3, CD34, CD45, iNOS, TNF-*α*, IL-1*β*, IL-2, IL-4, IL-6, IL-10, IL-12A, and IFN-*γ* were regarded as positive. The numbers of each immunolabeled cells, located in the spleen and S-LN parenchyma were counted by a computer [50, 53] with slight modification, respectively.

### 2.12. Statistical Analyses

All numerical data were expressed as mean ± standard deviation (SD). Multiple comparison tests for different dose groups were conducted. Variance homogeneity was examined using the Levene test [54]. If the Levene test indicated no significant deviations from variance homogeneity, the data were analyzed by one-way ANOVA test followed by the least-significant differences (LSD) multi-comparison test to determine which pairs of group comparisons were significantly different. In case of significant deviations from variance, homogeneity was observed at the Levene test, a nonparametric comparison test, Kruskal-Wallis H test was conducted. When a significant difference is observed in the Kruskal-Wallis H test, the Mann-Whitney U (MW) test was conducted to determine the specific pairs of group comparison, which are significantly different. Statistical analyses were conducted using SPSS for Windows (Release 14.0 K, IBM SPSS Inc., Armonk, NY, USA) [55]. In addition, the percent changes between normal and control groups at each sacrifice time were calculated to observe the severities of immune suppresses induced by treatment of morphine at dose levels of 0.1, 1.0, 5.0, and 10.0 mg/kg at each sacrifice times 3D, 7D, 21D, and 21D3DW, according to the previous studies [51, 56], respectively.

(1) $$\begin{array}{c} \text{Comparison with normal group}\left(\%\right)=\left[\left(\frac{\left(\text{Data of each morphine treated groups}-\text{Data of normal rats at equal sacrifice time}\right)}{\text{Data of normal rats at equal sacrifice time}}\right)\times 100\right]. \end{array}$$

In addition, the percent changes between equal dosages of i. v. and i. p. treatments were calculated to observe the differences along with the administration route, according to the previously established methods [51, 57], respectively.

(2) $$\begin{array}{c} \text{Comparison with equal dosage of }i.v.\left(\%\right)=\left[\frac{\left(\text{Data of equal dosage of }i.p-\text{Data of equal dosage of }i.v.\right)}{\text{Data of equal dosage of }i.v.}\right]\times 100. \end{array}$$

Also, the percent changes between normal and control groups were calculated to observe the severity of immune decrease induced by morphine, and between control and acupuncture groups to observe immunomodulatory effects of acupuncture as following equations (3) and (4), according to the previous studies [56, 57].

Equation (3) is given as follows

(3) $$\begin{array}{c} \text{Percentage changes as compared with normal group}\left(\%\right)=\left[\frac{\text{Data of control}-\text{Data of normal}}{\text{Data of normal}}\times 100\right]. \end{array}$$

Equation (4) that says Percentage changes as compared with control group (%) is shown as follows

(4) $$\begin{array}{c} \text{Percentage changes as compared with normal group}\left(\%\right)=\left[\frac{\left(\text{Data of acupuncture groups}-\text{Data of control}\right)}{\text{Data of control}}\times 100\right]. \end{array}$$

## 3. Results

### 3.1. Morphine Effect on Immunity

**Figure 1:**
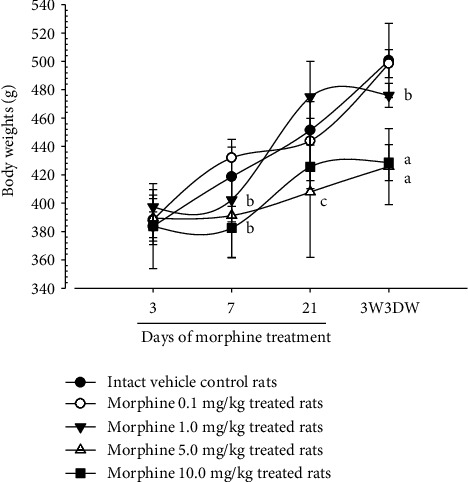
Body weight changes. Significant decreases of body weights were shown in M 1.0 group at 12D3DW and M 5.0 and 10.0 groups from 7 days as compared to intact vehicle control (normal) group at each sacrifice time, respectively. Values are expressed as Mean ± SD. ^a^*p* < 0.01 and ^b^*p* < 0.05 by LSD test as compared to normal group at equal sacrifice time. ^c^*p* < 0.01 by MW test as compared to normal group at equal sacrifice time.

**Figure 2:**
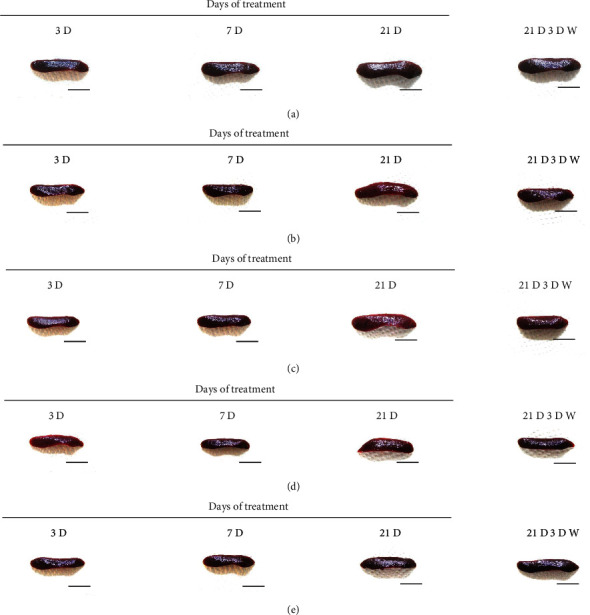
Representative gross Spleen images. Significant (*p* < 0.01 or *p* < 0.05) increases of semiquantitative spleen atrophic scores were observed in M 0.1, 1.0, 5.0 and 10.0 groups from 3 days as compared to normal group at equal sacrifice time, respectively. No dose-dependent increases of gross spleen atrophic changes were demonstrated between M 5.0 and 10.0 groups, and similar spleen gross findings were observed in 21D3DW as compared to those of 21D. (a) normal; (b) M 0.1; (c) M 1.0; (d) M 5.0; (e) M 10.0. Scale bar: 16.50 mm.

**Figure 3:**
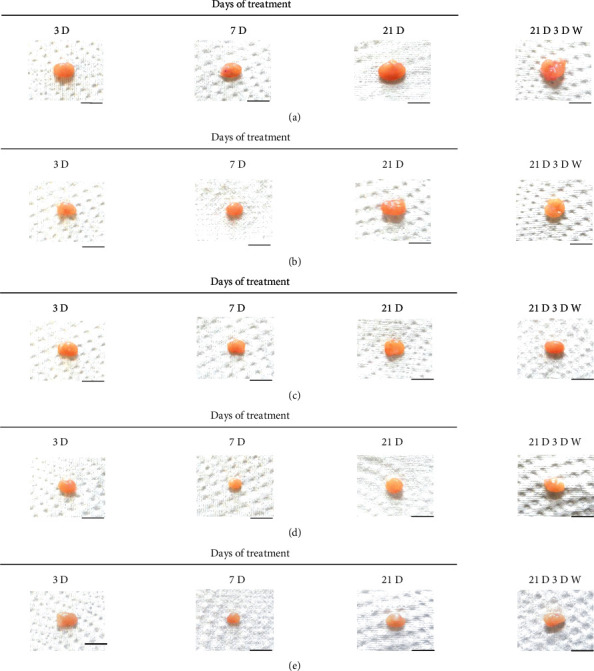
Representative gross S-LN images. Significant (*p* < 0.01 or *p* < 0.05) increases of semiquantitative S-LN atrophic scores were observed in M 0.1 and 1.0 groups at 21D and in 5.0 and 10.0 groups from 7 days as compared to normal group at equal sacrifice time, respectively. No dose-dependent increases of gross S-LN atrophic changes were demonstrated between M 5.0 and 10.0 groups, and similar S-LN gross findings were observed in 21D3DW as compared to those of 21D. (a) normal; (b) M 0.1; (c) M 1.0; (d) M 5.0; (e) M 10.0. Scale bar: 6 mm.

**Figure 4:**
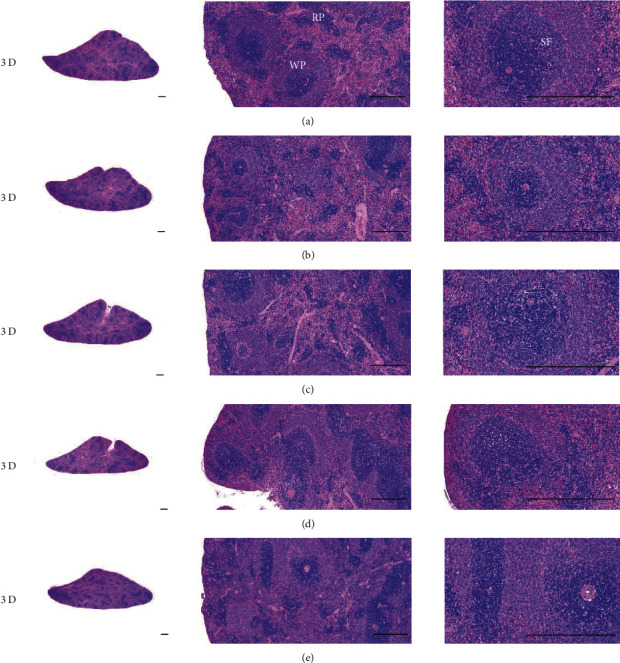
Representative histological Spleen images at 3D. Decrease of total thickness, white pulp diameter and numbers in spleen were demonstrated in all M groups. No dose-dependent increases of histopathological spleen atrophic changes were demonstrated between M 5.0 and 10.0 at 3D. (a) normal; (b) M 0.1; (c) M 1.0; (d) M 5.0; (e) M 10.0. All hematoxylin and eosin stain. Scale bar: 400 *μ*m. WP : White pulp; RP : Red pulps; SF : Secondary follicle.

**Figure 5:**
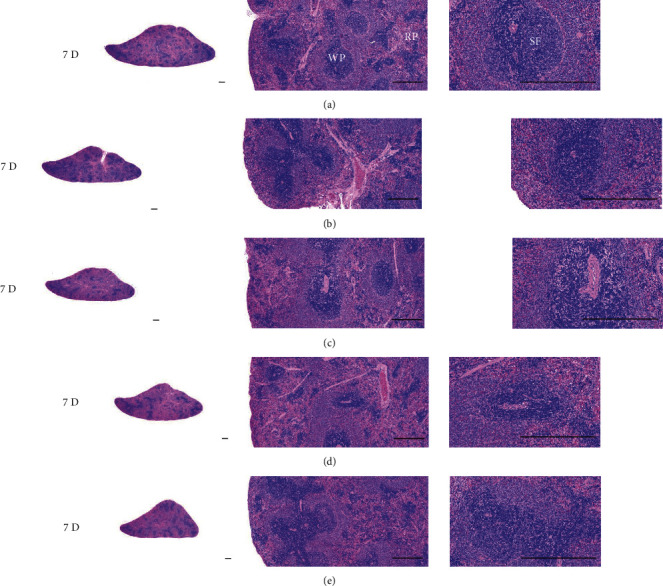
Representative histological Spleen images at 7D. Decrease of total thickness, white pulp diameter and numbers in spleen were demonstrated in all M groups. No dose-dependent increases of histopathological spleen atrophic changes were demonstrated between M 5.0 and 10.0 at 7D. (a) normal; (a) M 0.1; (c) M 1.0; (d) M 5.0; (e) M 10.0. All hematoxylin and eosin stain. Scale bar: 400 *μ*m. WP : White pulp; RP : Red pulps; SF : Secondary follicle.

**Figure 6:**
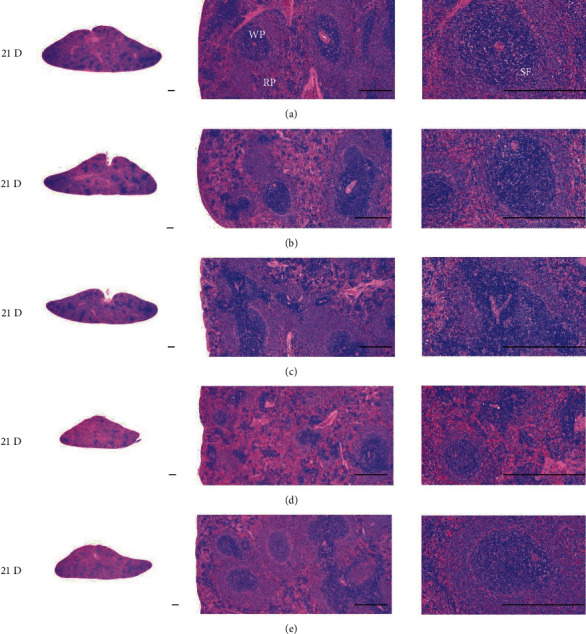
Representative histological Spleen images at 21D. Decrease of total thickness, white pulp diameter and numbers in spleen were demonstrated in all M groups. No dose-dependent increases of histopathological spleen atrophic changes were demonstrated between M 5.0 and 10.0 at 7D. (a) normal; (b) M 0.1; (c) M 1.0; (d) M 5.0; (e) M 10.0. All hematoxylin and eosin stain. Scale bar: 400 *μ*m. WP : White pulp; RP : Red pulps; SF : Secondary follicle.

**Figure 7:**
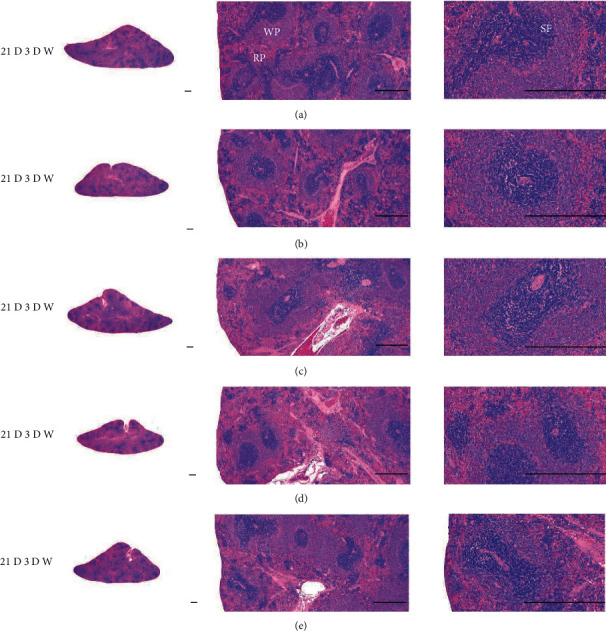
Representative histological Spleen images at 21D3DW. Decrease in total thickness, white pulp diameter and numbers in spleen were demonstrated in all M groups. No dose-dependent increases of histopathological spleen atrophic changes were demonstrated between M 5.0 and 10.0 at 21D3DW. (a) normal; (b) M 0.1; (c) M 1.0; (d) M 5.0; (e) M 10.0. All hematoxylin and eosin stain. Scale bar: 400 *μ*m. WP : White pulp; RP : Red pulps; SF : Secondary follicle.

**Figure 8:**
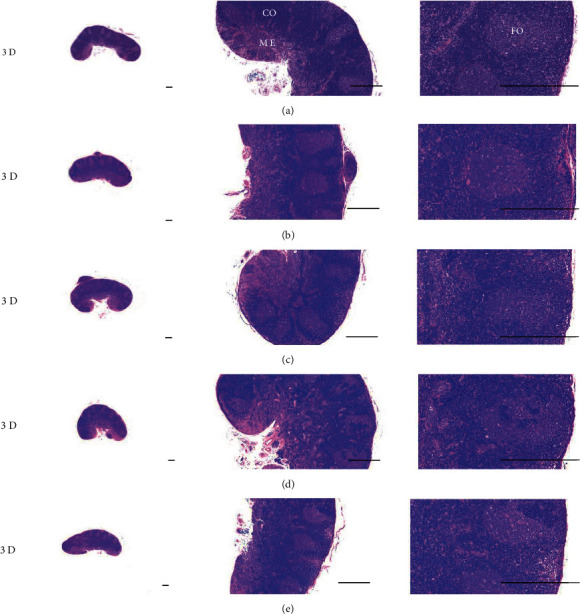
Representative histological S-LN (Submandibular lymph node) images at 3D. No significant or meaningful S-LN atrophic changes–changes on the total and cortex thicknesses, and lymphoid follicle numbers of S-LN were demonstrated in all M groups. (a) normal; (b) M 0.1; (c) M 1.0; (d) M 5.0; (e) M 10.0. All hematoxylin and eosin stain. Scale bar: 400 *μ*m. CO: cortex; ME: medullar; FO: follicle.

**Figure 9:**
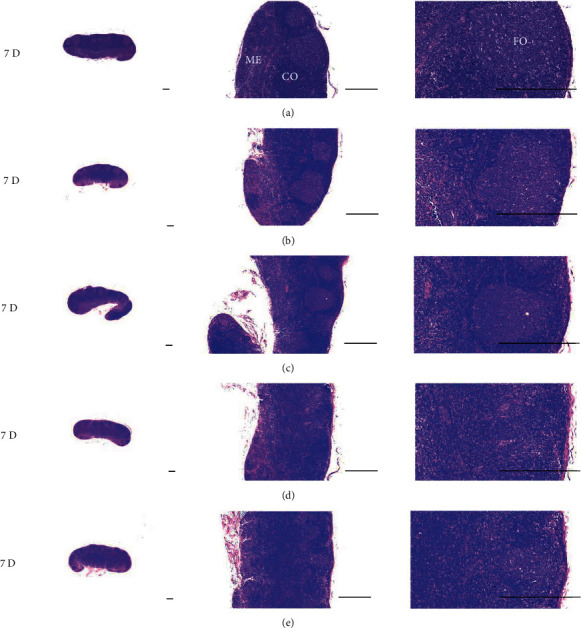
Representative histological S-LN images at 7D. Although no significant or meaningful S-LN atrophic changes-changes in the total and cortex thicknesses, and lymphoid follicle numbers of S-LN were shown in M 0.1 and 1.0 groups compared to normal, noticeable S-LN atrophic changes, significant decreases of the total and cortex thicknesses, and lymphoid follicle numbers of S-LN were demonstrated in M 5.0 and 10.0 groups. No dose-dependent increases of histopathological S-LN atrophic changes were demonstrated between M 5.0 and 10.0. (a) normal; (b) M 0.1; (c) M 1.0; (d) M 5.0; (e) M 10.0. All hematoxylin and eosin stain. Scale bar: 400 *μ*m. CO: cortex; ME: medullar; FO: follicle.

**Figure 10:**
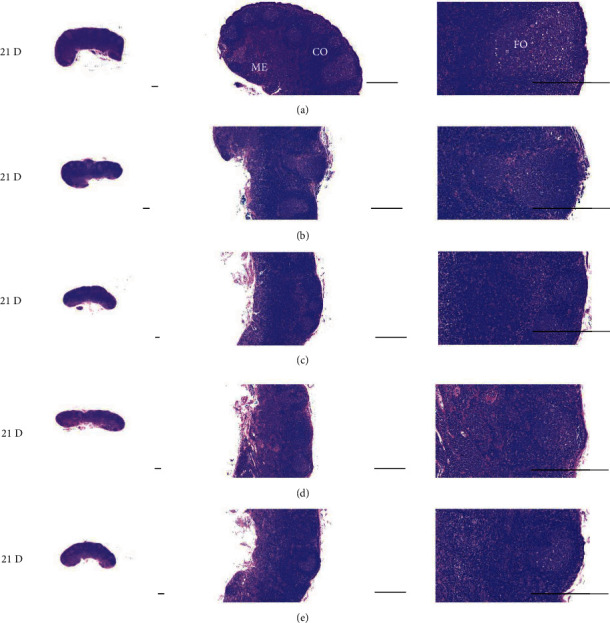
Representative histological S-LN Images at 21D. Although no significant or meaningful S-LN atrophic changes–changes on the total and cortex thicknesses, and lymphoid follicle numbers of S-LN were demonstrated in M 0.1 as compared to normal, noticeable S-LN atrophic changes, significant decreases of the total and cortex thicknesses, and lymphoid follicle numbers of S-LN were demonstrated in M 1.0, 5.0 and 10.0 groups. No dose-dependent increases of histopathological S-LN atrophic changes were demonstrated between morphine 1.0, 5.0 and 10.0. (a) normal; (b) M 0.1; (c) M 1.0; (d) M 5.0; (e) M 10.0. All hematoxylin and eosin stain. Scale bar: 400 *μ*m. CO: cortex; ME: medullar; FO: follicle.

**Figure 11:**
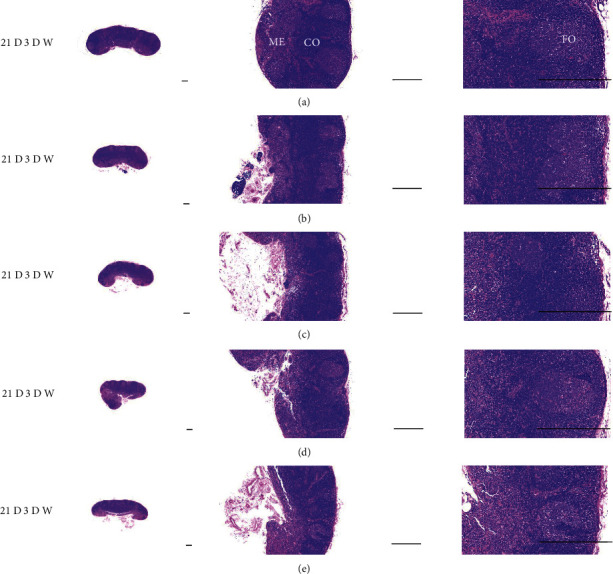
Representative histological S-LN Images at 21D3DW. Although no significant or meaningful S-LN atrophic changes-changes on the total and cortex thicknesses, and lymphoid follicle numbers of S-LN were demonstrated in M 0.1 group as compared to normal, noticeable S-LN atrophic changes, significant decreases of the total and cortex thicknesses, and lymphoid follicle numbers of S-LN were demonstrated in M 1.0, 5.0 and 10.0 groups. No dose-dependent increases of histopathological S-LN atrophic changes were demonstrated between M 1.0, 5.0 and 10.0 groups, and similar histopathological S-LN atrophic findings were observed at 21D3DW as compared to those of 21D. (a) normal; (b) M 0.1; (c) M 1.0; (d) M 5.0; (e) M 10.0. All hematoxylin and eosin stain. Scale bar: 400 *μ*m. CO: cortex; ME: medullar; FO: follicle.

Although M 1.0 resulted in lower body weight at 21D3DW, both M 5.0 and M 10.0 reduced body weight change at 7D, 21D, and 21D3DW (Figure 1, Table 2). Decreases of absolute and relative spleen and S-LN weights, increases in gross and histopathological atrophic changes (increases of gross semiquantitative scores, decrease of total thickness, white pulp diameter and numbers of spleen, total and cortex thicknesses, and lymphoid follicle numbers of S-LN at histomorphometric analysis) were demonstrated as morphine-induced immune suppression signs in M 5.0 and 10.0 at 21D and 21D3DW (Figures 2–11, Tables 3-7). However, no dose-dependent increases of immunosuppress signs were demonstrated between M 5.0 and M 10.0, also similar spleen and S-LN atrophic changes were observed in 21D3DW as compared to 21D.

### 3.2. Comparison between Administration Routes

**Figure 12:**
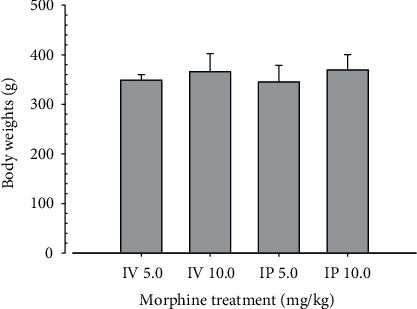
Body weight changes. No significant changes on the body weights were demonstrated in M 5 and 10 i. p. rats as compared to those of equal dosage of i. v. rats. Values are expressed as Mean ± SD.

**Figure 13:**
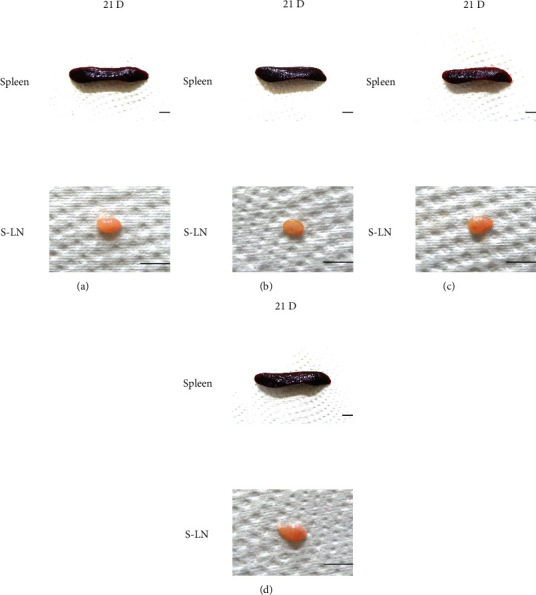
Representative gross Spleen and S-LN images. No meaningful changes on the spleen and S-LN gross findings were demonstrated in M 5 and 10 i. p. rats as compared to those of i. v. (a) M 5, i. v.; (b) M 10, i. v.; (c) M 5, i. p.; (d) M 10, i. p. Scale bars = 9 mm.

**Figure 14:**
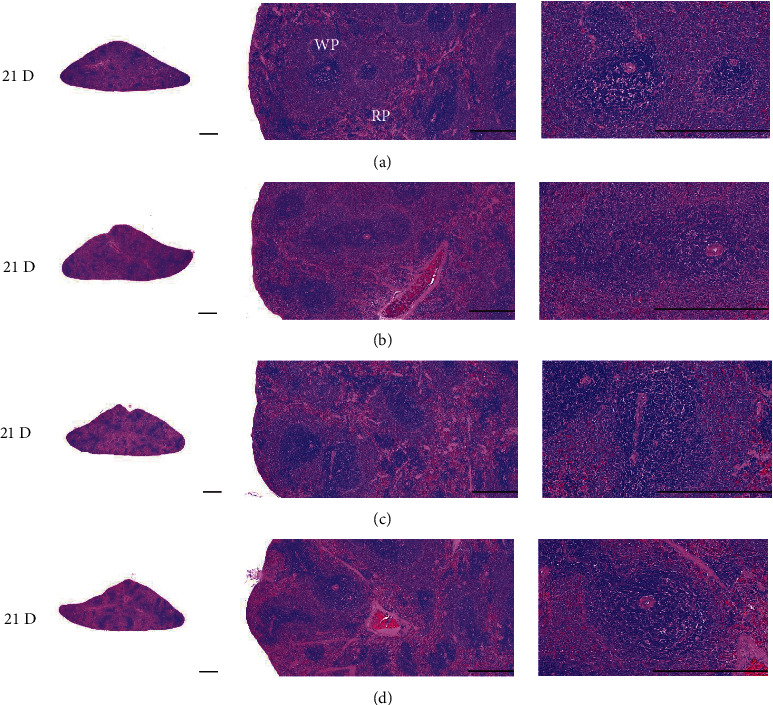
Representative histological Spleen images. No significant changes on the spleen histopathological findings were demonstrated in M 5 and 10 i.p. rats as compared to those of i.v. (a) M 5, i.v.; (b) M 10, i.v.; (c) M 5, i.p.; (d) M 10, i.p. All hematoxylin and eosin stain. Scale bars = 400 *μ*m. WP = White pulp; RP = Red pulps.

**Figure 15:**
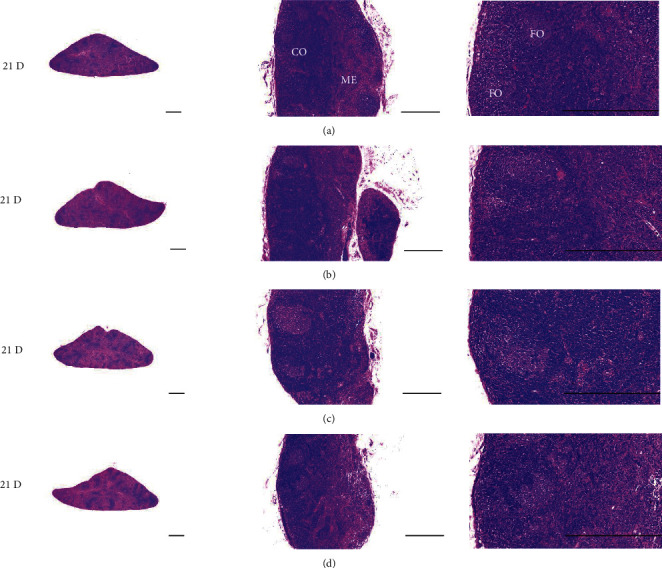
Representative histological S-LN Images. No meaningful changes on the S-LN histopathological findings were demonstrated in M 5 and 10 rats as compared to those of i.v. All hematoxylin and eosin stains. Scale bars = 400 *μ*m. CO = cortex; ME = medullar; FO = follicle.

According to the results of the first experiment, we selected M 5.0 and M 10.0 and 21D as proper conditions to induce immune suppression and investigated if administration routes of i.v. and i. p. results in difference. After i. v. or i. p. treatment with morphine for 21D, the body weights at sacrifice, spleen and S-LN weights, and gross and histopathological findings were evaluated. Total thickness, white pulp diameter and numbers of spleen, total and cortex thicknesses, and lymphoid follicle numbers of S-LN were measured as histomorphometric items. The results were compared between equal dosages of i. v. or i. p. administration. The changes in body and organ weights showed no significant difference between i. v. and i. p. (Figure 12, Table 8). The gross and histopathological findings showed similar results (Figures 13–15, Tables 9-11), suggesting that morphine-induced immunosuppress occurred regardless of administration routes of i.v. or i.p.

### 3.3. Acupuncture Effects on Morphine-Induced Immune Suppression

In the present study, we have observed a possibility of immune modulation by acupuncture. Following the first and second experiments, M 10 was administered for 21D intravenously and the body weight, spleen and left S-LN weights, and splenic NK cell activities were observed with histopathological findings (total thickness, white pulp diameter of spleen, total and cortex thicknesses, and lymphoid follicle numbers of S-LN), and CD3, CD4, CD8, Foxp3, CD34, CD45, IL-1*β*, IL-2, IL-4, IL-6, IL-10, IL-12A, iNOS, TNF-*α,* and IFN-*γ* immunoreactive cell numbers in the spleen and S-LN parenchyma (positive cells/mm^2^) were measured.

**Figure 16:**
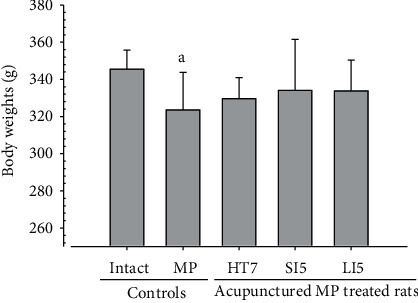
Body Weights. In the present study, significant decreases of body weights were detected in morphine control group as compared to normal (intact) group; however, no significant changes on the body weights were demonstrated in all acupuncture groups as compared to morphine control group. Values are expressed as Mean ± SD. MP: morphine. ^a^*p* < 0.05 as compared to normal (intact) by LSD test.

**Figure 17:**
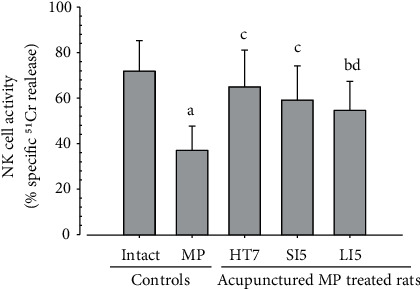
Splenic NK cell activities. Significant decrease of splenic NK cell activity was observed in morphine control group as compared to normal (intact) group, however this decrease was reversed by HT7, SI5 and LI5 acupunctures, in that orders. Values are expressed as Mean ± SD. MP: morphine; NK : Natural killer. ^a^*p* < 0.01 and ^b^*p* < 0.05 as compared to normal group, ^c^*p* < 0.01 and ^d^*p* < 0.05 as compared to morphine control group by LSD test.

**Figure 18:**
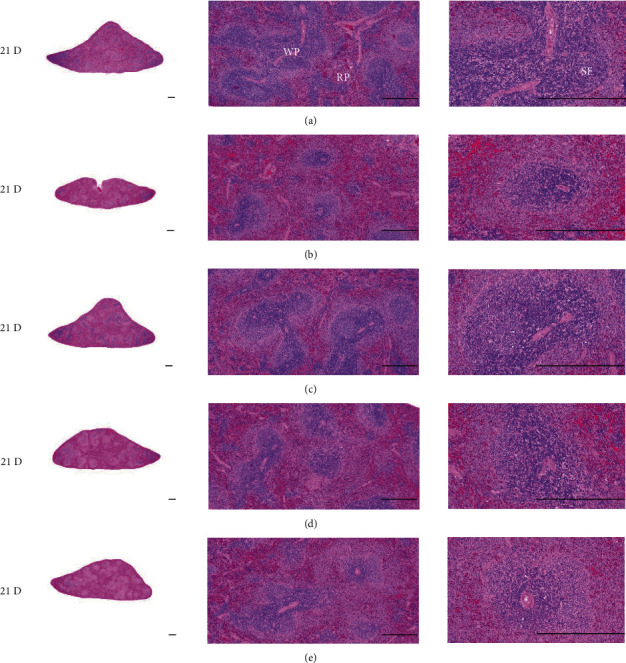
Representative gross histological Spleen images. In morphine control group, significant decreases of total spleen thickness, white pulp diameter and numbers of spleen were demonstrated as compared to those of normal. However, these morphine-induced changes were obviously and significantly normalized by HT7, SI5, and LI5 acupunctures, in that orders. (a) normal; (b) M 10; (c) M 10 + HT7 acupuncture; (d) M 10 + SI5 acupuncture; (e) M 10 + LI5 acupuncture. All hematoxylin and eosin stain. Scale bar: 400 *μ*m. WP : White pulp; RP : Red pulps; SF : Secondary follicle.

**Figure 19:**
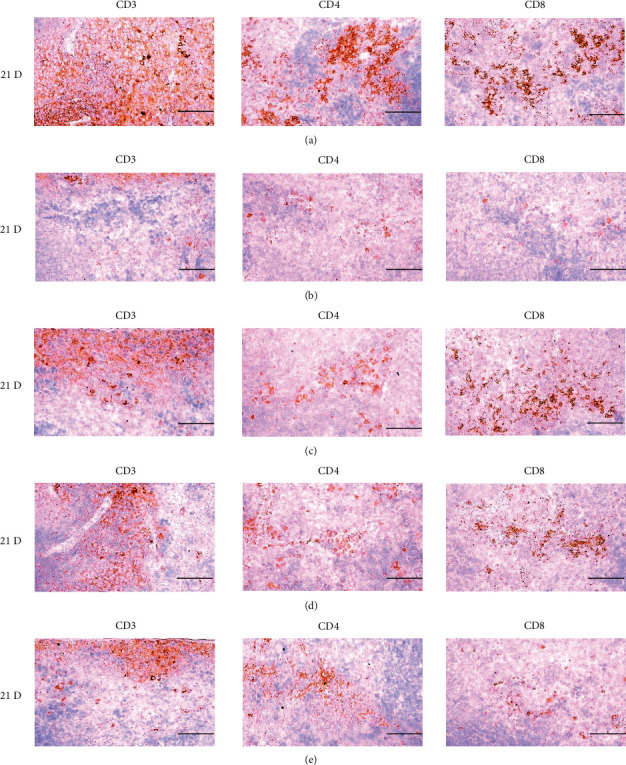
Representative images of CD3, CD4 and CD8 immunoreactive cells in the Spleen. In morphine control group, significant decreases of splenic cells immunolabeled for CD3 (general T cell marker), CD4 (help T cell marker) and CD8 (cytotoxic T cell marker) were demonstrated as compared to those of normal group, however, these morphine-inducedimmunosuppress-related decreases of CD3, CD4 and CD8 immunoreactivity were obviously and significantly normalized by HT7, SI5 and LI5 acupunctures, in that orders. (a) normal; (b) M 10; (c) M 10 + HT7 acupuncture; (d) M 10 + SI5 acupuncture; (e) M 10 + LI5 acupuncture. All ABC based immunohistochemistric eosin stain. Scale bar: 80 *μ*m. CD : Cluster of differentiation; ABC : Avidin-biotin-peroxidase complex.

**Figure 20:**
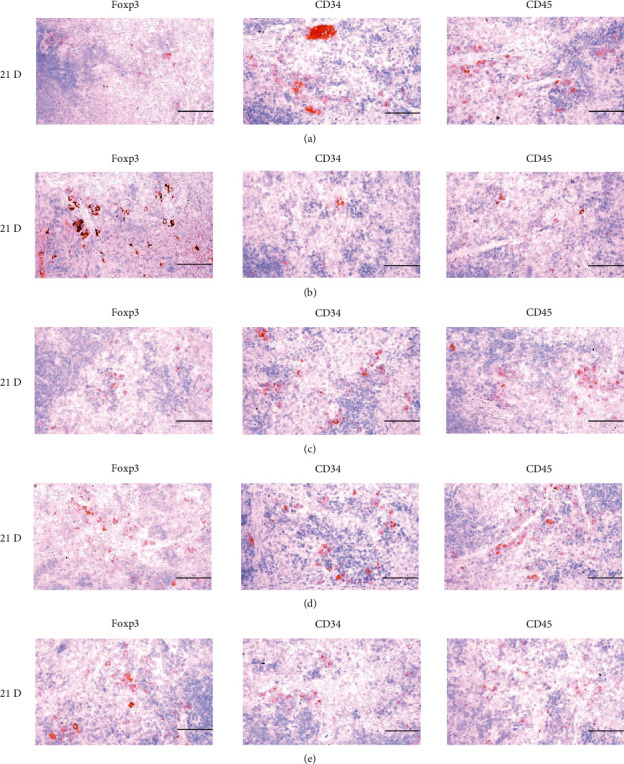
Representative images of Foxp3, CD34 and CD45 Immunoreactive cells in the Spleen. In morphine control group, significant increases of splenic cells immunolabeled for Foxp3 (regulatory T cell marker), and decreases of CD34 (general stem cell marker) and CD45 (hematopoietic stem cell marker) were demonstrated as compared to those of normal group, however these morphine-inducedimmunosuppress-related increases of Foxp3 immunoreactivity, and decreases of CD34 and CD45 immunoreactivity were obviously and significantly normalized by HT7, SI5 and LI5 acupunctures, in that orders. (a) normal; (b) M 10; (c) M 10 + HT7 acupuncture; (d) M 10 + SI5 acupuncture; (e) M 10 + LI5 acupuncture. All ABC based immunohistochemistric eosin stain. Scale bar: 80 *μ*m. Foxp3: Forkhead box P3; CD : Cluster of differentiation; ABC : Avidin-biotin-peroxidase complex.

**Figure 21:**
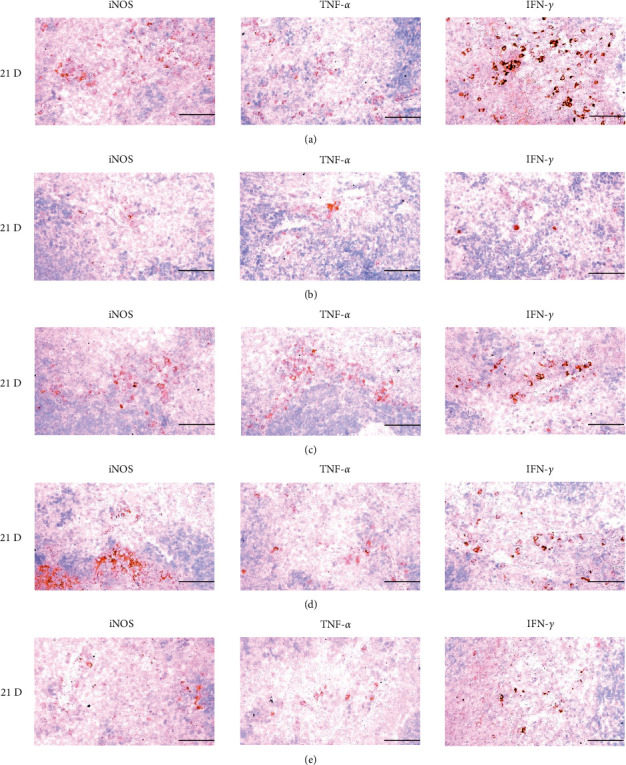
Representative images of iNOS, TNF-*α* and IFN-*γ* immunoreactive cells in the Spleen. In morphine control group, significant decreases of splenic immune stimulatory cytokines-iNOS, TNF-*α* and IFN-*γ* immunolabeled cells were demonstrated as compared to those of normal group, however these decreases of iNOS, TNF-*α* and IFN-*γ* immunoreactivity were obviously and significantly normalized by HT7, SI5 and LI5 acupunctures, in that orders. (a) normal; (b) M 10; (c) M 10 + HT7 acupuncture; (d) M 10 + SI5 acupuncture; (e) M 10 + LI5 acupuncture. All ABC-based immunohistochemistric eosin stain. Scale bar: 80 *μ*m. iNOS : Inducible nitric oxide synthase; TNF : Tumor necrosis factor; IFN : Interferon; ABC : Avidin-biotin-peroxidase complex.

**Figure 22:**
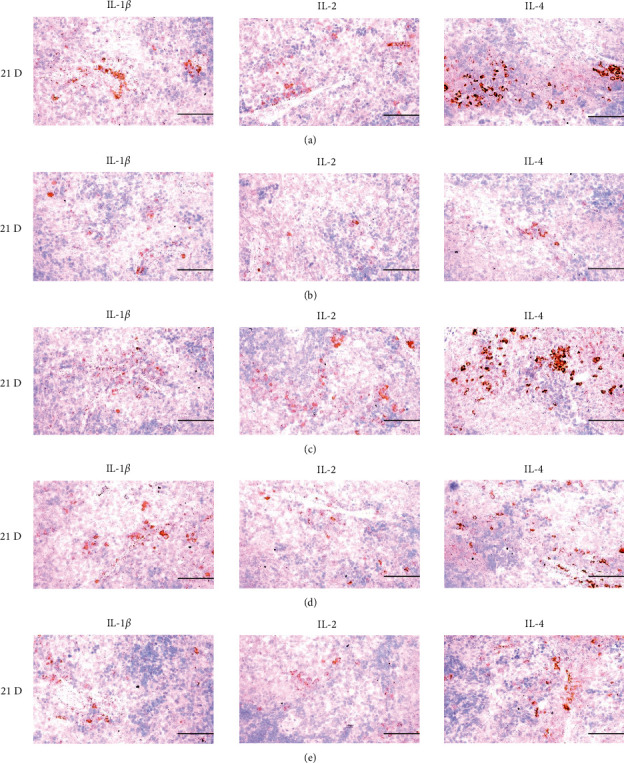
Representative images of IL-1*β*, IL-2, and IL-4 immunoreactive cells in the Spleen. In morphine control group, significant decreases of splenic immune stimulatory cytokines-IL-1*β*, IL-2 and IL-4 immunolabeled cells were demonstrated as compared to normal group, however, these morphine-induced decreases of IL-1*β*, IL-2, and IL-4 immunoreactivity were obviously and significantly normalized by HT7, SI5, and LI5 acupunctures, in that orders. (a) normal; (b) M 10; (c) M 10 + HT7 acupuncture; (d) M 10 + SI5 acupuncture; (e) M 10 + LI5 acupuncture. All ABC-based immunohistochemistric eosin stain. Scale bars: 80 *μ*m. IL : Interleukin; ABC : Avidin-biotin-peroxidase complex.

**Figure 23:**
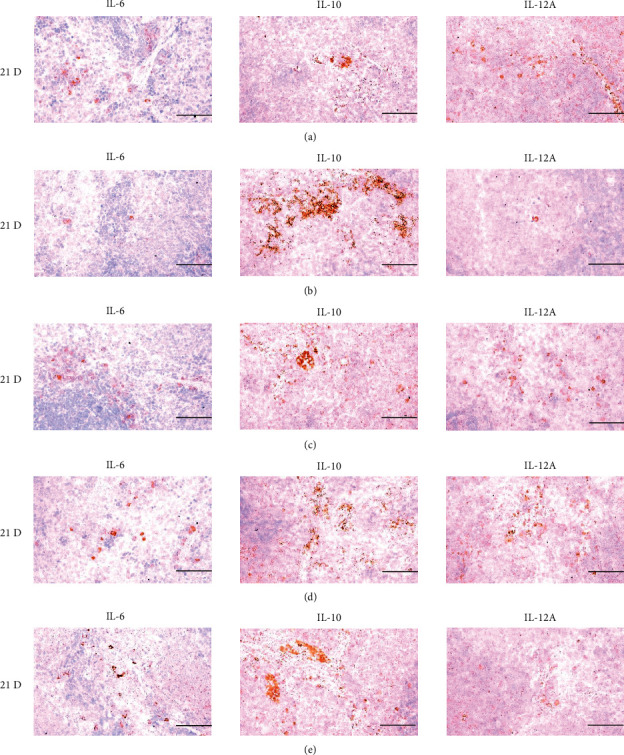
Representative images of IL-6, IL-10, and IL-12A immunoreactive cells in the Spleen. In morphine control group, significant decreases of splenic immune stimulatory cytokines-IL-6 and IL-12A immunolabeled cells, and increase of immune suppressive cytokine-IL-10 immunolabeled cells on the spleen were demonstrated as compared to those of normal group, however these morphine-induced decreases of IL-6 and IL-12, and increases of IL-10 immunoreactivity were obviously and significantly normalized by HT7, SI5 and LI5 acupunctures, in that orders. (a) normal; (b) M 10; (c) M 10 + HT7 acupuncture; (d) M 10 + SI5 acupuncture; (e) M 10 + LI5 acupuncture. All ABC-based immunohistochemistric eosin stain. Scale bars: 80 *μ*m. IL : Interleukin; ABC : Avidin-biotin-peroxidase complex.

**Figure 24:**
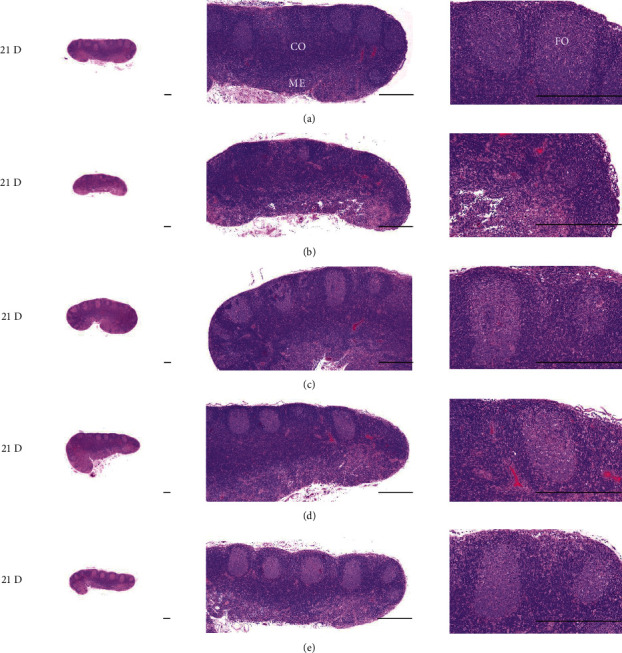
Representative gross histological S-LN Images. In morphine control group, significant total and cortex S-LN thicknesses, and lymphoid follicle numbers of S-LN were demonstrated as compared to those of normal, suggesting morphine-induced immunosuppression. However, these morphine-induced histopathological changes were clearly and significantly normalized by HT7, SI5, and LI5 acupunctures, in that orders. (a) normal; (b) M 10; (c) M 10 + HT7 acupuncture; (d) M 10 + SI5 acupuncture; (e) M 10 + LI5 acupuncture. All hematoxylin and eosin stain. Scale bars: 400 *μ*m. S-LN : Submandibular lymph node; CO: cortex; ME; medullar; FO: follicle.

**Figure 25:**
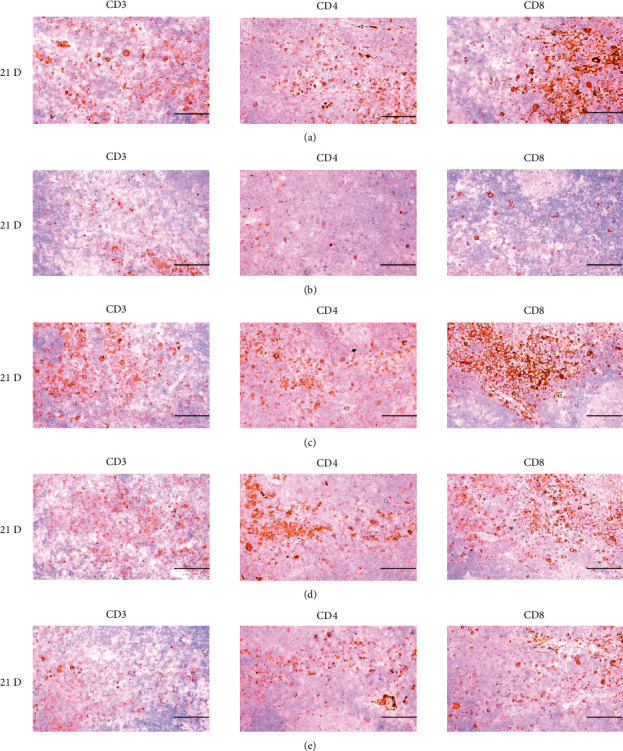
Representative images of CD3, CD4 and CD8 immunoreactive cells in the S-LN. In morphine group, significant decreases of S-LN CD3 (general T cell marker), CD4 (help T cell marker) and CD8 (cytotoxic T cell marker) immunolabeled cells were demonstrated as compared to those of normal group, however these morphine-induced decreases of CD3, CD4 and CD8 immunoreactive were obviously and significantly normalized by HT7, SI5, and LI5 acupunctures, in that orders. (a) normal; (b) M 10; (c) M 10 + HT7 acupuncture; (d) M 10 + SI5 acupuncture; (e) M 10 + LI5 acupuncture. All ABC-based immunohistochemistric eosin stain. Scale bars: 80 *μ*m. S-LN : Submandibular lymph node; CD : Cluster of differentiation; ABC : Avidin-biotin-peroxidase complex.

**Figure 26:**
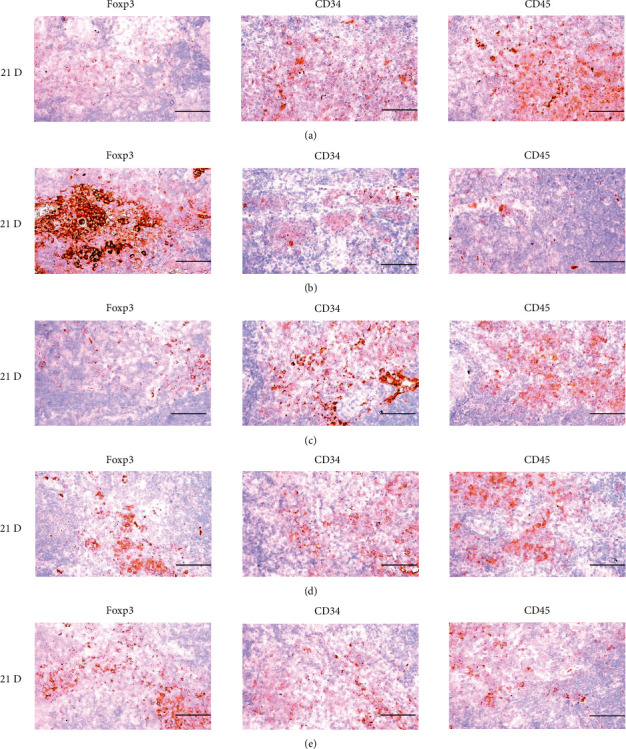
Representative images of Foxp3, CD34 and CD45 immunoreactive cells in the S-LN. In morphine group, significant increases of S-LN Foxp3 (regulatory T cell marker), and decreases of CD34 (general stem cell marker) and CD45 (hematopoietic stem cell marker) immunolabeled cells were demonstrated as compared to those of normal, however, these morphine-induced increases of Foxp3 and decreases of CD34 and CD45 immunoreactivity cells were obviously and significantly normalized by HT7, SI5 and LI5 acupunctures, in that orders. (a) normal; (b) M 10; (c) M 10 + HT7 acupuncture; (d) M 10 + SI5 acupuncture; (e) M 10 + LI5 acupuncture. All ABC based immunohistochemistric eosin stain. Scale bars: 80 *μ*m. S-LN : Submandibular lymph node; Foxp3: Forkhead box P3; CD : Cluster of differentiation; ABC : Avidin-biotin-peroxidase complex.

**Figure 27:**
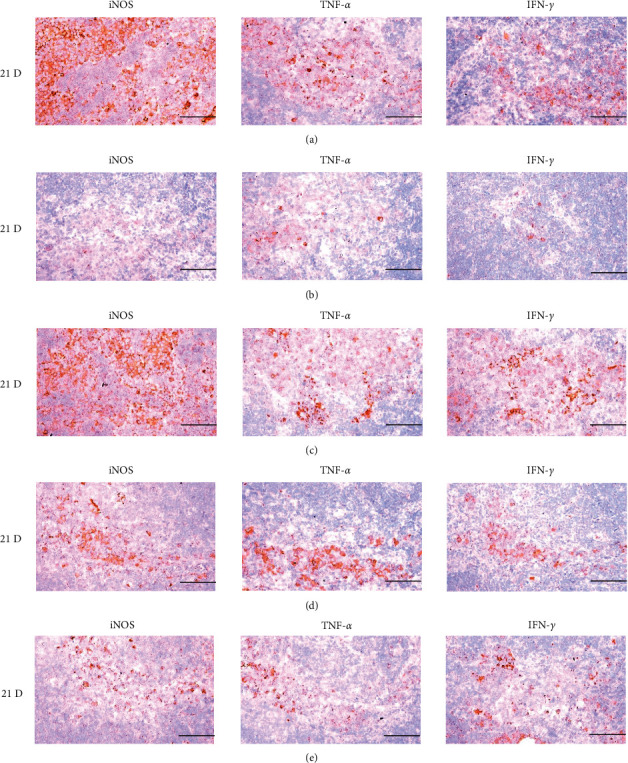
Representative images of iNOS, TNF-*α,* and IFN-*γ* immunoreactive cells in the S-LN. In morphine group, significant decreases of S-LN immune stimulatory cytokines-iNOS, TNF-*α* and IFN-*γ* immunolabeled cells were demonstrated as compared to those of normal, however, these morphine-induced decreases of iNOS, TNF-*α* and IFN-*γ* immunoreactivity were obviously and significantly normalized by HT7, SI5, and LI5 acupunctures, in that orders. (a) normal; (b) M 10; (c) M 10 + HT7 acupuncture; (d) M 10 + SI5 acupuncture; (e) M 10 + LI5 acupuncture. All ABC-based immunohistochemistric eosin stain. Scale bars: 80 *μ*m. S-LN : Submandibular lymph node; iNOS : Inducible nitric oxide synthase; TNF : Tumor necrosis factor; IFN : Interferon; ABC : Avidin-biotin-peroxidase complex.

**Figure 28:**
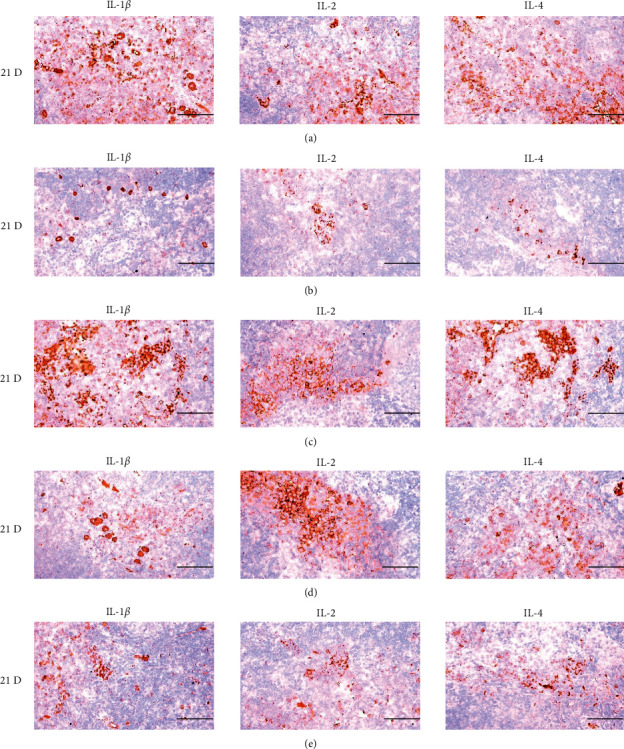
Representative images of IL-1*β*, IL-2, and IL-4 immunoreactive cells in the S-LN. In morphine group, significant decreases of S-LN immune stimulatory cytokines-IL-1*β*, IL-2 and IL-4 immunolabeled cells were demonstrated as compared to those of normal, however these morphine-induced decreases of IL-1*β*, IL-2 and IL-4 immunoreactivity were obviously and significantly normalized by HT7, SI5 and LI5 acupunctures, in that orders. (a) normal; (b) M 10; (c) M 10 + HT7 acupuncture; (d) M 10 + SI5 acupuncture; (e) M 10 + LI5 acupuncture. All ABC-based immunohistochemistric eosin stain. Scale bars: 80 *μ*m. S-LN : Submandibular lymph node; IL : Interleukin; ABC : Avidin-biotin-peroxidase complex.

**Figure 29:**
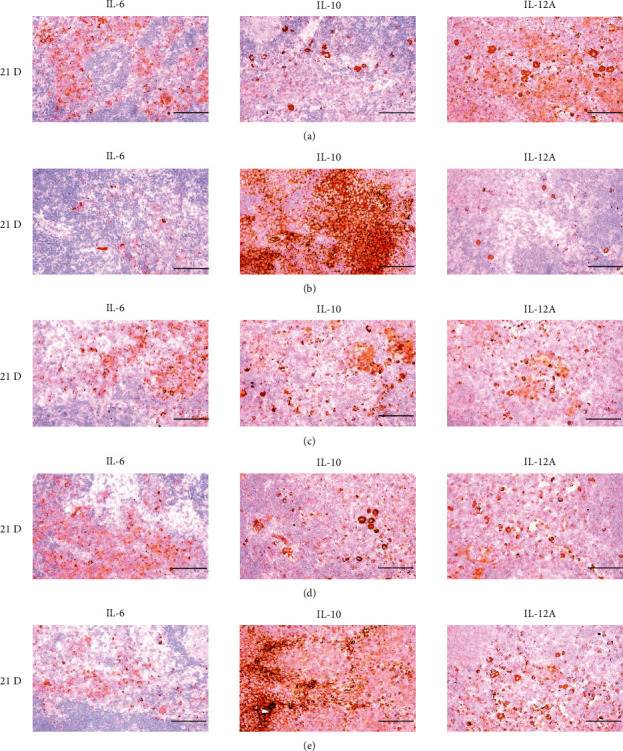
Representative images of IL-6, IL-10, and IL-12A immunoreactive cells in the S-LN. In morphine group, significant decreases of S-LN immune stimulatory cytokines-IL-6 and IL-12A immunolabeled cells, and increase of immune suppressive cytokine-IL-10 immunolabeled cells on the S-LN were demonstrated as compared to those of normal, however these morphine-induced decreases of IL-6 and IL-12, and increase of IL-10 immunoreactivity were obviously and significantly normalized by HT7, SI5 and LI5 acupunctures, in that orders. (a) normal; (b) M 10; (c) M 10 + HT7 acupuncture; (d) M 10 + SI5 acupuncture; (e) M 10 + LI5 acupuncture. All ABC based immunohistochemistric eosin stain. Scale bars: 80 *μ*m. S-LN : Submandibular lymph node; IL : Interleukin; ABC : Avidin-biotin-peroxidase complex.

In morphine control rats, significant decreases of body weights, spleen and left S-LN absolute and relative weights, splenic NK cell activities, total spleen thickness, white pulp diameter and numbers of spleen, total and cortex S-LN thicknesses, lymphoid follicle numbers of S-LN, CD3, CD4, CD8, CD34, CD45, iNOS, TNF-*α*, IL-1*β*, IL-2, IL-4, IL-6, IL-12A, and IFN-*γ* immunolabeled cell numbers, and increases of Foxp3 and IL-10 immunoreactivity were demonstrated compared to normal group, showing morphine-induced immunosuppresses. However, these morphine-induced immunosuppresses were obviously and significantly normalized by HT7, SI5, and LI5 acupuncture, in that order (Figures 16–29, Tables 12-14).

## 4. Discussion

The present study confirmed the morphine-induced immune suppression in Sprague-Dawley rats. Vehicle or Morphine was treated, and rats were sacrificed at 3, 7, 21D including 21D3DW. The body weights at sacrifice, spleen and left S-LN weights, gross and histopathological findings were observed. Total thickness, white pulp diameter and numbers in spleen, total and cortex thicknesses, and lymphoid follicle numbers of S-LN were used as histomorphometric measures.

Decreased absolute and relative spleen and S-LN weights and increased gross and histopathological atrophic changes (increases of gross semiquantitative scores, decrease of the total thickness, white pulp diameter and numbers in spleen, total and cortex thicknesses, and lymphoid follicle numbers in S-LN at histomorphometric analysis) regarded as immunosuppress signs [50, 51] were induced by morphine, in parallel with other studies [1, 15]. These signs were demonstrated obviously and significantly, in particular, by M 5.0 and 10.0 at 21D in the first experiment. However, no dose-dependent increases in immunosuppress signs were demonstrated between M 5.0 and 10.0. Also, similar spleen and S-LN atrophic changes were observed at 21D3DW compared to those of 21D. These findings were considered as direct evidence that an appropriate morphine induced-immunosuppress rat model was produced by 21D treatment at a dose level of M 5.0 or M 10.0 and that 3D of withdrawal did not deteriorate morphine-induced immunosuppress, at least in the condition of the present experiment (Figures 2–11, Tables 3-7).

Morphine was reported to inhibit body weight increase in rats [58], and more seriously after short withdrawal [59, 60]. This is parallel with our results that more severe decreases in body weights were demonstrated at 21D3DW in M 1.0, 5.0, and 10.0 as compared to each dosage at 21D. Thus, in the first experiment, an appropriate morphine-induced-immune suppression rat model was confirmed by 21D treatment with M 5.0 and 10.0.

In the second experiment, we compared the morphine induced-immunosuppress between administration routes, (i. v. and i. p.) with M 5.0 and 10.0 treatment for 21D. After treatment, the body, spleen, and S-LN were weighed, and gross and histopathological findings were observed. Total thickness, white pulp diameter and numbers in spleen, total and cortex thicknesses, and lymphoid follicle numbers in S-LN were measured as histomorphometric analysis. The results were compared between equal dosages of i. v. and i. p. and showed that no significant or meaningful changes were demonstrated between i. v. or i. p at equal dosage (Figures 12–15, Tables 8–11), suggesting that morphine-induced immunosuppress regardless of administration route.

The third experiment was performed to examine the immunomodulatory potential of acupuncture and investigate a possible mechanism underlying the acupuncture effects. The appropriate morphine-induced immune suppression conditions were decided as 21D treatment with M 10.0 and i. v., based on the first and second experiments, and to mimic the human administration. Body weights at sacrifice, spleen and left S-LN weights, and splenic NK cell activities were observed as histopathological findings. Total thickness, white pulp diameter and numbers of spleen, total and cortex thicknesses, and lymphoid follicle numbers of S-LN, and CD3, CD4, CD8, Foxp3, CD34, CD45, IL-1*β*, IL-2, IL-4, IL-6, IL-10, IL-12A, iNOS, TNF-*α,* and IFN-*γ* immunoreactive cell numbers in the spleen and S-LN parenchyma (positive cells/mm^2^) were measured.

The decrease in body weight observed in the control group was not shown in the acupunctured rats (Figure 16). Decreases of absolute and relative spleen and S-LN weights, total thickness, white pulp diameter and numbers in spleen, total and cortex thicknesses, and lymphoid follicle numbers of S-LN at histomorphometric analysis are regarded as classic immunosuppress characteristics [50, 51] were demonstrated as morphine-induced immunosuppress in the control group, in parallel with other studies [1, 15, 17]. However, these morphine-induced immunosuppress signs were obviously and significantly normalized by HT7, SI5, and LI5 acupunctures, in that order. These findings are considered clear evidence that HT7, SI5, and LI5 acupuncture have potent and favorable inhibitory activities against morphine-induced atrophic changes in lymphoid organs (Figures 18 and 24, Tables 12–14).

NK cells are representative immune cells, and activation of NK cells has been highlighted as new treatment regimen for cancer and other immunosuppressive diseases [61, 62]. In this study, significant decreases in splenic NK cell activities were observed in control rats however they were normalized by HT7, SI5, and LI5 acupunctures (Figure 17), suggesting definitive immunomodulatory effects of acupuncture through splenic NK cell activations on morphine-induced immune suppression. This result is parallel with a previous study demonstrating that decreased postoperative NK cell activity induced by morphine was reversed by electro-acupuncture [63].

T cell antigen receptors are always membrane-bound and noncovalently associated with a set of four invariant glycoproteins collectively called CD3. Thus, CD3 has been regarded as a marker of T-cells [64]. CD4 is a single-chain glycoprotein of 55 kDa, and CD8 is a disulfide-linked heterodimer of a 34 kDa subunit. Either CD4 or CD8 is found on mature T cells, although immature T cells may express both. Their function is to determine the class MHC molecule that is recognized by a T cell. Generally, CD4+ cells are called helper T cell and CD8+ cells are cytotoxic T cell [65]. Foxp3, a protein involved in immune responses [66], is a member of the FOX protein family and appears to function as a master regulator of the regulatory pathway in the development and function of Treg cells [67]. Treg cells generally decrease the immune response. In cancer, an excess of regulatory T cell activity can prevent the immune system from destroying cancer cells. In autoimmune disease, a deficiency in regulatory T cell activity can allow other autoimmune cells to attack the body's own tissues [68, 69]. Foxp3 has been used as a valuable marker for Treg cell activity, and increments in Foxp3+ cells represent immune suppression [66, 67, 70]. In our immune-histochemistric analysis, significant decreases in CD3, CD4 and CD8+ cells, and increases in Foxp3+ cells were demonstrated in the control group, suggesting morphine-induced immunosuppresses. However, these morphine-inducedimmunosuppression-related changes in T cell subsets were normalized by acupunctures (Figures 19, 20, 25, and 26). These findings confirm that acupunctures have potent and favorable immunomodulatory activities against morphine induced-immune suppression through modulation of T cell subset.

The cytokine TNF-*α*, produced by a variety of cell types including splenocytes, is associated with critical events leading to T-lineage commitment and differentiation [71]. TNF-*α* can enhance the *in vivo* immune response at doses much lower than those that cause weight loss or tissue toxicity. It enhances the proliferation of B and T cells and promotes the generation of cytotoxic T cells. In addition, it enhances IL-2-induced immunoglobulin production and augments IL-2stimulated-natural killer cell activity and proliferation of monocytes [72]. IL-1 is another cytokine released to various cell types such as macrophages, dendritic cells, lymphocytes, endothelial cells, fibroblasts and keratocytes, and is necessary for the successful initiation of some forms of immune response [73]. IL-2 is a type of cytokine signaling molecule in the immune system. It is a 15.5–16 kDa protein [74] that regulates the activities of white blood cells (leukocytes, often lymphocytes) that are responsible for immunity [75]. IL-4 is a cytokine that induces the differentiation of naive helper T cells (Th0 cells) to Th2 cells. Upon activation by IL-4, Th2 cells subsequently produce additional IL-4 in a positive feedback loop. The cell that initially produces IL-4, thus inducing Th2 differentiation, has not been identified, however basophils may be the effector cell [76]. IL-10 is an immunosuppressive glycoprotein of 19–21 kDa that is secreted by Th2 cells, some B cells, and activated macrophages. IL-10 primarily acts on activated macrophages to suppress the secretion of IL-1, IL-12, TNF-*α*, and ROS [72]. IL-6 is secreted by Th2 cells and macrophages to stimulate immune response during infection and after trauma, leading to inflammation [77]. IL-6 also plays a role in fighting infection, as IL-6 has been shown to be required for resistance against the bacterium *Streptococcus pneumonia* [78]. IL-12 is naturally produced by dendritic cells, macrophages, neutrophils, and human B-lymphoblastoid cells in response to antigenic stimulation [79], and is involved in the differentiation of naive T cells into Th1 cells through stimulating the production of IFN-*γ* and TNF-*α* [80]. In addition, IFN-*γ* is a glycoprotein of 20 to 25 kDa produced by CD8+ T cells, Th1 cells, and NK cells in response to IL-2. It has complex effect on B and T cell functions and enhances the NK cell and macrophage activities [72]. In this study, significant decreases of spleen and S-LN iNOS, TNF-*α*, IL-1*β*, IL-2, IL-4, IL-6, IL-12A and IFN-*γ*+ cells, and increases of IL-10+ cells in the spleen and S-LN were demonstrated in the control group, suggesting morphine-induced immunosuppression. However, these morphine-inducedimmunosuppression-related cytokine changes were significantly normalized by HT7, SI5 and LI5 acupunctures, in those orders (Figures 21–23, Tables 12–14). These findings are considered reliable evidence that HT7, SI5, and LI5 acupunctures have potent and favorable immunomodulatory activities against morphine-induced immunosuppression, through cytokine normalization, at least in a condition of the present experiment.

CD34+ cells have been regarded as general stem cells, and CD45+ cells are considered hematopoietic stem cells [81, 82]. CD45 is a pan-leukocyte protein with tyrosine phosphatase activity involved in the regulation of signal transduction in hematopoiesis [83]. CD45 has been used as a valuable pan-leukocyte marker [84]. In our results, significant decreases of CD34 and CD45+ cells were demonstrated in the spleen and S-LN of control rats, as courses of immune suppression. However, these morphine-inducedimmunosuppression-related decreases of the CD35 and CD45+ cells were significantly inhibited by HT7, SI5, and LI5 acupuncture (Figures 20 and 26). These findings are considered direct evidence that HT7, SI5, and LI5 acupuncture have immunomodulatory activities against morphine-induced immunosuppression, through stem cell migration and differentiation to immune cells.

Taken together, the histopathological changes and the abnormal cytokines and immune cell activities induced by morphine were normalized by acupuncture.

Morphine weakens the immune system and suppresses immune response in the spleen, thymus, and lymph nodes [1, 4]. Opioid decreases the number of NK cells [18, 19] and B and T lymphocytes [20, 21]. A possible mechanism through which morphine induces immune decrease is that morphine acts as a potential oxidative stress inducer [7, 11–13]. Nutrients supporting the immune system are antioxidant providers and immune cells such as T-cells, NK cells, and T-helper cells have high levels of ROS. Also, immune cell membranes have a lot of polyunsaturated fatty acids, susceptible to ROS-related damage [26, 28]. Therefore, nutrients with antioxidant property extinguish the free radicals and regulate the immune system [26, 29].

In previous studies, acupuncture showed antioxidant properties [42–44], especially in morphine-induced liver injury [13], and regulated ROS levels [45]. Given that morphine is a potential oxidative stressor and weakens the immune system [1, 4, 15] and that antioxidation is important to support immune system, we suggest that acupunctures' inhibitory action against morphine induced-immune suppression probably is mediated, at least in part, via antioxidation.

## 5. Conclusion

Our results showed that the appropriate morphine induced-immunosuppress rat model was confirmed by 21D treatment with doses of 5.0 and 10.0. Also, no dose-dependent increases of immunosuppress signs were demonstrated between M 5.0 and 10.0, and 3 days of withdrawal was not influenceable. In addition, the immunosuppress by morphine was induced regardless of administration routes (i. v. or i. p.).

Most importantly, the key immune parameters–spleen and S-LN weights, splenic NK cell activities, and the T cell subsets (CD3, CD4, CD8, and Foxp3), general and hematopoietic stem cells (CD34 and CD45), and major immune-related cytokines (iNOS, TNF-*α*, IL-1*β*, IL-2, IL-4, IL-6, IL-12A, and IFN-*γ*) immunopositive cells showed immunosuppress signs by morphine, however, these morphine-inducedimmunosuppress-related signs were normalized by acupunctures, suggesting that acupuncture can be a new potent alternative immunomodulatory remedy for immune disorders by morphine. Further studies are needed to elucidate a more dedicated mechanism underlying acupuncture effects.

## Figures and Tables

**Table 1:** Primary antisera and detection kits are used in the immunohistochemistric stain.

Antisera or detection kits	Code	Source	Dilution
Primary antisera^*∗*^			
Anti-CD3 (PC3/188A) antibody	Sc-20047	Santa cruz biotechnology, santa cruz, CA, USA	1 : 100
Anti-CD4 (H-370) antibody	Sc-7219	Santa cruz biotechnology, santa cruz, CA, USA	1 : 100
Anti-CD8 antibody	ab4055	Abcam, cambridge, UK	1 : 100
Anti-mouse/Rat Foxp3 (FJK-16 s) antibody	17-5773-82	Thermo Fisher scientific, rockford, IL, USA	1 : 100
Anti-CD34 antibody	ab185732	Abcam, cambridge, UK	1 : 100
Anti-CD45 antibody	ab10558	Abcam, cambridge, UK	1 : 100
Anti-IL-1*β* (H-153) antibody	Sc-7884	Santa cruz biotechnology, santa cruz, CA, USA	1 : 100
Anti-IL-2 antibody	NBP2-16948	Novus biologicals, centennial, CO, USA	1 : 100
Anti-IL-4 antibody	ab9811	Abcam, cambridge, UK	1 : 100
Anti-IL-6 antibody	NB600-1131	Novus biologicals, centennial, CO, USA	1 : 100
Anti-IL-10 antibody	ab192271	Abcam, cambridge, UK	1 : 100
Anti-IL-12A antibody	ab203031	Abcam, cambridge, UK	1 : 100
Anti-interferongamma Antibody	ab216642	Abcam, cambridge, UK	1 : 100
Anti-NOS2 (N-20) antibody	Sc-651	Santa cruz biotechnology, santa cruz, CA, USA	1 : 100
Anti-TNF-*α* (4E1) antibody	Sc-130349	Santa cruz biotechnology, santa cruz, CA, USA	1 : 100

Detection kits			
Vectastain elite ABC kit	PK-6200	Vector lab. Inc., CA, USA	1 : 50
Peroxidase substrate kit	SK-4100	Vector lab. Inc., CA, USA	1 : 50

^*∗*^All antisera were diluted using 0.01 M phosphate-buffered saline. CD: Cluster of differentiation; Foxp3: Forkhead box P3; IL: nterleukin; NOS2: Inducible nitric oxide synthase, iNOS; TNF: Tumor necrosis factor.

**Table 2:** Body weights (*g*).

Groups	Days of morphine treatment			21D3DW
	3D	7D	21D	
Vehicle	383.60 ± 10.36	418.60 ± 20.86	451.40 ± 8.38	500.60 ± 26.23
M 0.1	388.20 ± 17.41	432.00 ± 13.04	443.80 ± 27.82	498.40 ± 9.91
M 1.0	397.40 ± 12.24	402.40 ± 15.50	475.00 ± 24.98	476.00 ± 8.43^b^
M 5.0	389.40 ± 13.78	391.20 ± 29.70^b^	407.80 ± 46.02^a^	425.80 ± 26.76^a^
M 10.0	383.80 ± 29.99	382.40 ± 20.60^b^	425.60 ± 15.39^c^	428.60 ± 12.62^a^

Values are expressed as Mean ± SD. ^a^*p* < 0.01 and ^b^*p* < 0.05 as compared with equal sacrifice time of vehicle by LSD test. ^c^*p* < 0.01 as compared with equal sacrifice time of vehicle by MW test.

**Table 3:** Absolute weights (*g*) of spleen and left S-LN.

Groups	Days of morphine treatment			21D3DW
	3D	7D	21D	
Spleen				
Vehicle	0.851 ± 0.030	0.868 ± 0.097	1.355 ± 0.091	1.309 ± 0.042
M 0.1	0.729 ± 0.038^b^	0.688 ± 0.014^a^	1.097 ± 0.124^a^	0.966 ± 0.072^a^
M 1.0	0.747 ± 0.026^b^	0.688 ± 0.036^a^	1.089 ± 0.123^a^	0.961 ± 0.068^a^
M 5.0	0.744 ± 0.037^b^	0.675 ± 0.048^a^	0.912 ± 0.098^a^	0.891 ± 0.116^a^
M 10.0	0.740 ± 0.063^b^	0.662 ± 0.043^a^	0.951 ± 0.102^a^	0.929 ± 0.072^a^

Left S-LN				
Vehicle	0.034 ± 0.011	0.036 ± 0.004	0.042 ± 0.002	0.053 ± 0.007
M 0.1	0.035 ± 0.008	0.035 ± 0.003	0.025 ± 0.005^a^	0.032 ± 0.010^c^
M 1.0	0.033 ± 0.009	0.034 ± 0.002	0.018 ± 0.005^a^	0.030 ± 0.004^b^
M 5.0	0.034 ± 0.004	0.023 ± 0.003^a^	0.016 ± 0.003^a^	0.021 ± 0.003^b^
M 10.0	0.035 ± 0.005	0.020 ± 0.005^a^	0.019 ± 0.007^a^	0.025 ± 0.003^b^

Values are expressed as Mean ± SD. ^a^*p* < 0.01 as compared with equal sacrifice time of vehicle by LSD test.

**Table 4:** Relative weights of spleen and left S-LN.

Groups	Days of morphine treatment			21D3DW
	3D	7D	21D	
Spleen				
Vehicle	0.222 ± 0.008	0.207 ± 0.021	0.300 ± 0.023	0.262 ± 0.012
M 0.1	0.188 ± 0.014^a^	0.160 ± 0.008^a^	0.249 ± 0.038^b^	0.194 ± 0.015^a^
M 1.0	0.188 ± 0.012^a^	0.171 ± 0.007^a^	0.229 ± 0.021^a^	0.202 ± 0.015^a^
M 5.0	0.191 ± 0.009^a^	0.173 ± 0.012^a^	0.227 ± 0.041^a^	0.210 ± 0.033^a^
M 10.0	0.193 ± 0.009^a^	0.173 ± 0.013^a^	0.224 ± 0.030^a^	0.217 ± 0.022^a^

Left S-LN				
Vehicle	0.009 ± 0.003	0.009 ± 0.001	0.009 ± 0.000	0.011 ± 0.001
M 0.1	0.009 ± 0.002	0.008 ± 0.001	0.006 ± 0.001^a^	0.006 ± 0.002^a^
M 1.0	0.008 ± 0.002	0.008 ± 0.001	0.004 ± 0.001^a^	0.006 ± 0.001^a^
M 5.0	0.009 ± 0.001	0.006 ± 0.001^a^	0.004 ± 0.001^a^	0.005 ± 0.001^a^
M 10.0	0.009 ± 0.001	0.005 ± 0.001^a^	0.004 ± 0.002^a^	0.006 ± 0.001^a^

^b^ *p* < 0.01 and ^*c*^*p* < 0.05 as compared with equal sacrifice time of vehicle by MW test.  Table 4. Relative weights of spleen and left S-LN. ^a^*p* < 0.01 and ^b^*p* < 0.05 as compared with equal sacrifice time of vehicle by LSD test.

**Table 5:** Gross atrophic semiquantitative grading of spleen and left S-LN.

Groups	Days of morphine treatment			21D3DW
	3D	7D	21D	
Spleen				
Vehicle	0.20 ± 0.45	0.20 ± 0.45	0.00 ± 0.00^*∗*^	0.00 ± 0.00
M 0.1	1.20 ± 0.45^a^	1.20 ± 0.45^c^	1.00 ± 0.00^b^	1.00 ± 0.00^b^
M 1.0	1.00 ± 0.00^a^	1.20 ± 0.45^c^	1.20 ± 0.45^b^	1.20 ± 0.45^b^
M 5.0	1.00 ± 0.00^a^	1.60 ± 0.55^c^	1.40 ± 0.55^b^	1.60 ± 0.55^b^
M 10.0	1.20 ± 0.45^a^	1.40 ± 0.55^c^	1.40 ± 0.55^b^	1.20 ± 0.45^b^

Left S-LN				
Vehicle	0.20 ± 0.45	0.20 ± 0.45	0.00 ± 0.00	0.20 ± 0.45
M 0.1	0.20 ± 0.45	0.20 ± 0.45	0.80 ± 0.45^c^	1.00 ± 0.00^c^
M 1.0	0.20 ± 0.45	0.20 ± 0.45	1.20 ± 0.45^b^	1.00 ± 0.00^c^
M 5.0	0.20 ± 0.45	1.00 ± 0.00^a^	1.40 ± 0.55^b^	1.40 ± 0.55^c^
M 10.0	0.00 ± 0.00	1.20 ± 0.45^a^	1.20 ± 0.84^c^	1.20 ± 0.45^c^

Values are expressed as Mean ± SD of scores (Max = 3). ^*∗*^ Not detected-normal appearance. ^a^*p* < 0.01 as compared with equal sacrifice time of vehicle by LSD test. ^*b*^*p* < 0.01 and ^c^*p* < 0.05 as compared with equal sacrifice time of vehicle by MW test.

**Table 6:** Histomorphometric analysis of spleen.

Groups	Days of morphine treatment			21D3DW
	3D	7D	21D	
Total thickness (mm/central regions)				
Vehicle	4.10 ± 0.14	4.09 ± 0.08	4.10 ± 0.16	4.10 ± 0.09
M 0.1	3.70 ± 0.19^a^	3.43 ± 0.14^a^	3.23 ± 0.16^a^	3.29 ± 0.10^a^
M 1.0	3.45 ± 0.19^a^	3.31 ± 0.19^a^	3.32 ± 0.10^a^	3.33 ± 0.14^a^
M 5.0	3.19 ± 0.17^a^	3.16 ± 0.14^a^	2.83 ± 0.15^a^	2.80 ± 0.15^a^
M 10.0	3.20 ± 0.09^a^	3.17 ± 0.11^a^	2.95 ± 0.16^a^	2.98 ± 0.11^a^

White pulp thickness (*μ*m/white pulp)				
Vehicle	706.55 ± 42.09	705.18 ± 42.96	687.90 ± 26.01	697.28 ± 30.91
M 0.1	547.30 ± 42.22^a^	532.58 ± 24.28^a^	446.55 ± 44.21^a^	454.27 ± 16.66^a^
M 1.0	532.63 ± 18.28^a^	515.31 ± 18.12^a^	442.77 ± 52.12^a^	446.89 ± 34.68^a^
M 5.0	485.02 ± 27.78^a^	450.40 ± 41.38^a^	323.96 ± 19.79^a^	329.96 ± 25.90^a^
M 10.0	479.87 ± 37.66^a^	457.21 ± 42.56^a^	330.80 ± 27.75^a^	350.48 ± 21.28^a^

White pulp numbers (white pulps/mm^2^ of spleen)				
Vehicle	37.80 ± 3.27	39.00 ± 2.24	42.20 ± 3.77	39.00 ± 3.87
M 0.1	23.40 ± 2.07^a^	24.00 ± 1.58^a^	17.20 ± 1.92^a^	16.80 ± 1.92^b^
M 1.0	21.60 ± 2.07^a^	20.00 ± 1.58^a^	18.20 ± 1.48^a^	19.00 ± 1.00^b^
M 5.0	21.00 ± 1.58^a^	22.00 ± 1.58^a^	14.40 ± 2.61^a^	15.00 ± 2.24^b^
M 10.0	22.00 ± 1.22^a^	21.00 ± 2.74^a^	15.00 ± 1.87^a^	17.00 ± 1.58^b^

Values are expressed as Mean ± SD of scores (Max = 3). ^a^*p* < 0.01 as compared with equal sacrifice time of vehicle by LSD test. ^b^*p* < 0.01 as compared with equal sacrifice time of vehicle by MW test.

**Table 7:** Histomorphometric analysis of S-LN.

Groups	Days of morphine treatment			21D3DW
	3D	7D	21D	
Total thickness (*μ*m/central regions)	1113.41 ± 142.76	1129.19 ± 53.23	1156.66 ± 105.9	1159.38 ± 69.64

Vehicle				
M 0.1	1132.50 ± 125.68	1088.63 ± 85.11	1111.84 ± 91.10	1118.78 ± 107.11
M 1.0	1104.74 ± 82.85	1120.17 ± 61.29	910.99 ± 80.23^a^	903.92 ± 110.56^a^
M 5.0	1111.84 ± 88.97	892.74 ± 62.27^a^	906.83 ± 23.61^a^	906.17 ± 27.75^a^
M 10.0	1159.86 ± 54.82	1001.64 ± 88.95^b^	925.58 ± 47.83^a^	889.43 ± 62.40^a^

Cortex thickness (*μ*m/central regions)				
Vehicle	768.11 ± 53.96	795.00 ± 64.86	758.38 ± 34.23	765.32 ± 31.39
M 0.1	765.40 ± 54.72	787.55 ± 27.03	748.72 ± 39.76	737.55 ± 27.02
M 1.0	733.74 ± 27.52	782.24 ± 41.44	667.50 ± 26.34^a^	634.36 ± 40.17^a^
M 5.0	781.04 ± 32.02	649.01 ± 39.73^a^	528.43 ± 47.33^a^	529.87 ± 25.81^a^
M 10.0	778.42 ± 22.51	684.76 ± 12.85^a^	542.17 ± 37.85^a^	527.81 ± 23.78^a^

Lymphatic follicle numbers (follicles/mm^2^ of S-LN)				
Vehicle	26.80 ± 3.70	26.60 ± 4.04	26.20 ± 1.30	25.60 ± 2.07
M 0.1	26.40 ± 1.14	24.20 ± 1.64	25.40 ± 2.30	24.00 ± 1.58
M 1.0	26.40 ± 1.14	24.60 ± 2.07	16.60 ± 2.07^a^	17.80 ± 1.92^a^
M 5.0	25.60 ± 1.14	17.80 ± 1.92^a^	14.40 ± 1.14^a^	13.20 ± 1.30^a^
M 10.0	26.40 ± 1.14	17.40 ± 2.07^a^	13.60 ± 1.82^a^	12.60 ± 1.52^a^

Values are expressed as Mean ± SD of scores (Max = 3). ^a^*p* < 0.01 and ^b^*p* < 0.05 as compared with equal sacrifice time of vehicle by LSD test.

**Table 8:** Absolute and relative Spleen and left S-LN weights.

Groups	Spleen		S-LN	
	Absolute (g)	Relative (%)	Absolute (g)	Relative (%)
Intravenous				
M 5.0	0.870 ± 0.089	0.249 ± 0.017	0.023 ± 0.005	0.007 ± 0.001
M 10.0	0.837 ± 0.103	0.230 ± 0.035	0.020 ± 0.005	0.005 ± 0.002

Intraperitoneal				
M 5.0	0.842 ± 0.109	0.246 ± 0.039	0.023 ± 0.005	0.007 ± 0.002
M 10.0	0.871 ± 0.159	0.236 ± 0.044	0.020 ± 0.003	0.006 ± 0.001

Values are expressed as Mean ± SD.

**Table 9:** Gross atrophic semiquantitative grading systems of Spleen and left S-LN.

Organs	Intravenous		Intraperitoneal	
	M 5.0	M 10.0	M 5.0	M 10.0
Spleen	1.20 ± 0.45	1.50 ± 0.55	1.40 ± 0.55	1.33 ± 0.52
S-LN	1.20 ± 0.45	1.33 ± 0.52	1.20 ± 0.45	1.33 ± 0.52

Values are expressed as Mean ± SD. Sores, Max = 3.

**Table 10:** Histomorphometric analysis of Spleen.

Groups	Total thickness (*μ*m/central regions)	White pulp thickness (*μ*m/white pulp)	White pulps (numbers/mm^2^)
Intravenous			
M 5.0	3463.75 ± 384.14	406.88 ± 23.90	23.40 ± 3.71
M 10.0	3435.66 ± 343.53	391.67 ± 20.80	22.67 ± 2.16

Intraperitoneal			
M 5.0	3389.79 ± 318.73	404.29 ± 17.42	23.60 ± 2.88
M 10.0	3491.26 ± 273.84	402.00 ± 20.81	23.17 ± 3.06

Values are expressed as Mean ± SD.

**Table 11:** Histomorphometric analysis of S-LN.

Groups	Total thickness (*μ*m/central regions)	Cortex thickness (*μ*m/central regions)	Lymphoid follicles (numbers/mm^2^)
Intravenous			
M 5.0	1061.93 ± 162.62	603.42 ± 104.96	9.40 ± 1.14
M 10.0	972.74 ± 66.96	532.12 ± 76.26	9.33 ± 1.21

Intraperitoneal			
M 5.0	1049.58 ± 114.78	600.29 ± 66.73	9.80 ± 1.30
M 10.0	994.76 ± 55.94	548.91 ± 52.09	9.50 ± 1.05

Values are expressed as Mean ± SD.

**Table 12:** Absolute and relative Spleen and left S-LN weights.

Groups	Spleen		Left S-LN	
	Absolute (g)	Relative (%)	Absolute (g)	Relative (%)
Controls				
Normal	0.831 ± 0.052	0.241 ± 0.014	0.032 ± 0.007	0.009 ± 0.002
Morphine	0.651 ± 0.071^a^	0.201 ± 0.022^a^	0.016 ± 0.005^a^	0.005 ± 0.001^a^

Acupunctures				
HT7	0.790 ± 0.072^c^	0.240 ± 0.028^c^	0.032 ± 0.006^c^	0.010 ± 0.002^c^
SI5	0.760 ± 0.057^bc^	0.228 ± 0.018^c^	0.031 ± 0.006^c^	0.009 ± 0.002^c^
LI5	0.742 ± 0.049^ac^	0.223 ± 0.015^d^	0.030 ± 0.005^c^	0.009 ± 0.002^c^

Values are expressed as Mean ± SD. ^a^*p* < 0.01 and ^b^*p* < 0.05 as compared with normal group; ^c^*p* < 0.01 and ^d^*p* < 0.05 as compared with morphine group by LSD test.

**Table 13:** Histomorphometric analysis of Spleen.

Items (Unit)	Controls		Acupuncture groups		
	Normal	Morphine	HT7	SI5	LI5
Total thickness (*μ*m)	4191.58 ± 3 65.56	2259.11 ± 3 03.52^a^	3868.91 ± 29 9.41^c^	3475.02 ± 3 43.93^ac^	3041.15 ± 407.30ac
White pulp thickness (*μ*m)	456.87 ± 38.90	276.50 ± ± 27.6^a^	426.47 ± 39.43	404.35 ± 25.2, 4ac	370.24 ± 38.25^ac^
White pulp numbers/mm2	49.00 ± 10.11	24.29 ± 4.31^e^	42.78 ± 7.76^g^	38.44 ± 3.36^fg^	34.56 ± 4.33^eg^

Immunolabeled cells (positive cells/mm^2^)					
CD3	1208.33 ± 1, 84.94	380.00 ± 48, .83^e^	850.11 ± 98.2, 5eg	763.67 ± 86, .00^eg^	557.56 ± 82.50^e^, g
CD4	518.00 ± 84.52	198.86 ± 29.5, 3a	452.00 ± 62.24^bc^	384.78 ± 68.5, 9ac	341.89 ± 57.94^ac^
CD8	363.56 ± 51.8, 1	82.00 ± 25.46^a^	350.00 ± 31.67^c^	281.56 ± 34.6, 5ac	210.22 ± 71.57^ac^
Foxp3	30.00 ± 10.95	96.29 ± 13.83^a^	36.44 ± 10.38^c^	49.33 ± 12.29^a,^ c	63.78 ± 14.05^ac^
CD34	90.22 ± 17.16	10.57 ± 3.60^a^	86.67 ± 18.33^c^	79.11 ± 10.64^c^	47.33 ± 18.38^ac^
CD45	92.22 ± 11.72	23.14 ± 8.71^a^	76.89 ± 12.93^ac^	68.67 ± 10.10^a^, c	46.22 ± 10.27^ac^
iNOS	264.67 ± 77.2, 6	40.86 ± 18.58^a^	221.56 ± 62.82^c^	167.56 ± 78.8, 7ac	116.89 ± 50.90^ad^
TNF-*α*	121.11 ± 47.47	42.86 ± 4.88^e^	106.44 ± 19.51^g^	83.33 ± 10.63^f,^ g	59.56 ± 15.93^eg^
IL-1*β*	123.33 ± 31.2, 7	35.71 ± 10.67^a^	101.67 ± 18.72^bc^	88.67 ± 19.36^a,^ c	59.56 ± 11.44^ad^
IL-2	195.11 ± 46.25	27.71 ± 11.57^a^	190.89 ± 30.38^c^	120.67 ± 31.6, 5ac	80.44 ± 17.20^ac^
IL-4	237.56 ± 42.2, 0	36.00 ± 13.06^a^	225.11 ± 68.78^c^	172.89 ± 25.1, 6ac	124.67 ± 26.27^ac^
IL-6	84.67 ± 12.88	11.00 ± 4.20^e^	84.87 ± 21.02^g^	76.22 ± 10.74^g^	57.33 ± 15.49^eg^
IL-10	22.67 ± 9.27	264.57 ± 31.4, 7e	84.00 ± 14.35^eg^	134.89 ± 33.1, 8eg	185.67 ± 39.08^eg^
IL-12A	245.33 ± 42.8, 3	77.71 ± 21.43^a^	217.78 ± 38.36^c^	186.33 ± 36.0, 5ac	157.33 ± 20.78^ac^
IFN-*γ*	149.56 ± 25.0, 0	8.29 ± 4.39^e^	78.00 ± 11.70^eg^	72.22 ± 16.11^e,^ g	43.11 ± 10.01^eg^

Values are expressed as Mean ± SD. CD : Cluster of differentiation; Foxp3: Forkhead box P3; IL : Interleukin; NOS2 : Inducible nitric oxide synthase, iNOS; TNF : Tumor necrosis factor; IFN : Interferon. ^a^*p* < 0.01 and ^b^*p* < 0.05 as compared to normal group; ^c^*p* < 0.01 and ^d^*p* < 0.05 as compared to morphine group by LSD test. ^e^*p* < 0.01 and ^f^*p* < 0.05 as compared to normal group; ^g^*p* < 0.01 as compared with MP control by MW test.

**Table 14:** Histomorphometric analysis of left S-LN.

Items (Unit)	Controls		Acupuncture groups		
	Normal	Morphine	HT7	SI5	LI5
Total thickness (*μ*m)	1210.61 ± 152.88	747.33 ± 11	747.33 ± 11	747.33 ± 11	747.33 ± 11
Cortex thickness (*μ*m)	858.99 ± 121 .76	451.54 ± 68.2 3a	820.32 ± 111.51 c	746.99 ± 96.61^bc^	681.75 ± 96.64^ac^
Follicle numbers/mm2	23.67 ± 4.42	8.57 ± 1.99^a^	18.11 ± 3.62^ac^	14.44 ± 2.51^ac^	12.56 ± 2.07^ad^

Immunolabeled cells (positive cells/mm^2^)					
CD3	787.44 ± 17362	362.43 ± 59.8 1a	752.89 ± 116.01 c	555.78 ± 92.44^ac^	490.44 ± 60.76^ad^
CD4	524.33 ± 11267	196.43 ± 23.8 2a	510.67 ± 106.71 c	442.11 ± 95.96^c^	337.11 ± 86.61^ac^
CD8	362.22 ± 73 37	95.43 ± 19.72^a^	356.89 ± 54.01^c^	306.44 ± 81.44^c^	272.67 ± 38.04^ac^
Foxp3	120.67 ± 34 09	355.86 ± 72.8 9e	184.22 ± 38.65^eg^	204.22 ± 26.20^eg^	233.44 ± 56.25^eg^
CD34	497.11 ± 57 91	95.43 ± 17.61^a^	421.33 ± 59.60^ac^	321.89 ± 58.95^ac^	264.89 ± 68.24^ac^
CD45	460.78 ± 79.22	85.71 ± 16.14^a^	397.11 ± 85.89^c^	319.89 ± 101.02ac	250.44 ± 76.22^ac^
iNOS	686.00 ± 163.58	89.71 ± 12.46^e^	484.22 ± 76.88^eg^	305.67 ± 59.25^eg^	246.00 ± 78.14^eg^
TNF-*α*	430.00 ± 44.63	134.57 ± 30.57a	441.22 ± 51.08^c^	304.22 ± 41.33^ac^	215.33 ± 61.31^ac^
IL-1*β*	589.89 ± 114.95	114.00 ± 20.43e	457.78 ± 104.85fg	313.44 ± 73.04^eg^	194.67 ± 27.37^eg^
IL-2	485.33 ± 107.58	196.57 ± 60.57a	442.11 ± 100.77c	406.00 ± 64.41^c^	330.67 ± 73.71^ac^
IL-4	466.67 ± 79.49	110.29 ± 26.01a	436.00 ± 48.27^c^	387.67 ± 61.81^ac^	246.67 ± 47.81^ac^
IL-6	240.67 ± 50.86	99.43 ± 20.94^a^	193.33 ± 59.46^bc^	169.56 ± 22.18^ac^	153.33 ± 31.00^ad^
IL-10	91.11 ± 18.92	451.43 ± 76.29e	172.44 ± 29.07^eg^	252.89 ± 44.22^eg^	331.33 ± 39.95^eg^
IL-12A	637.56 ± 131.19	86.14 ± 15.98^e^	450.89 ± 86.90^eg^	350.22 ± 56.48^eg^	315.33 ± 106.17^eg^
IFN-*γ*	215.44 ± 46.38	45.29 ± 14.27^a^	203.3 ± 29.50^c^	158.33 ± 25.34^ac^	119.33 ± 51.17^ac^

Values are expressed as Mean ± SD. S-LN : Submandibular lymph node; CD : Cluster of differentiation; Foxp3: Forkhead box P3; IL : Interleukin; NOS2 : Inducible nitric oxide synthase, iNOS; TNF : Tumor necrosis factor; IFN : Interferon. ^a^*p* < 0.01 and ^b^*p* < 0.05 as compared to normal group; ^c^*p* < 0.01 and ^d^*p* < 0.05 as compared to morphine group by LSD test. ^e^*p* < 0.01 and ^f^*p* < 0.05 as compared to normal group; ^g^*p* < 0.01 as compared to morphine group by MW test.

## Data Availability

The data supporting this study can be obtained from the corresponding author upon request.
